# Prophylactic ablation during cardiac surgery in patients without atrial fibrillation: a systematic review and meta-analysis of randomized trials

**DOI:** 10.1093/icvts/ivae195

**Published:** 2024-11-26

**Authors:** Mika’il Visanji, Emilie P Belley-Côté, Ashok Pandey, Yael Amit, Graham R McClure, Jack Young, Kevin J Um, Alireza Oraii, Jeff S Healey, Richard P Whitlock, William F McIntyre

**Affiliations:** Department of Medicine, McMaster University, Hamilton, ON, Canada; Department of Medicine, McMaster University, Hamilton, ON, Canada; Population Health Research Institute, Hamilton, ON, Canada; Department of Pharmacology and Toxicology, University of Toronto, Toronto, ON, Canada; Department of Physiology, McGill University, Montreal, QC, Canada; Population Health Research Institute, Hamilton, ON, Canada; Department of Surgery, McMaster University, Hamilton, ON, Canada; Health Sciences Library, McMaster University, Hamilton, ON, Canada; Department of Medicine, McMaster University, Hamilton, ON, Canada; Population Health Research Institute, Hamilton, ON, Canada; Population Health Research Institute, Hamilton, ON, Canada; Department of Medicine, McMaster University, Hamilton, ON, Canada; Population Health Research Institute, Hamilton, ON, Canada; Population Health Research Institute, Hamilton, ON, Canada; Department of Surgery, McMaster University, Hamilton, ON, Canada; Department of Medicine, McMaster University, Hamilton, ON, Canada; Population Health Research Institute, Hamilton, ON, Canada

**Keywords:** Atrial fibrillation, Postoperative, Preventative, Electrophysiology, Arrhythmia

## Abstract

**OBJECTIVES:**

Atrial fibrillation is the most common complication of cardiac surgery and occurs frequently in patients without a history of the arrhythmia. We conducted a systematic review and meta-analysis of randomized controlled trials to assess whether prophylactic ablation during cardiac surgery in patients without a history of atrial fibrillation prevents atrial fibrillation.

**METHODS:**

We searched CENTRAL, MEDLINE and Embase from inception to August 2024. We included randomized trials of adults without a history of atrial fibrillation undergoing cardiac surgery. The intervention of interest was ablation during surgery. We pooled data using random-effects models. The primary outcome was new-onset early postoperative atrial fibrillation within 30 days following surgery. The key secondary outcome was incident clinical atrial fibrillation at follow-up (minimum 6 months). We assessed risk of bias using the Cochrane Collaboration’s risk of bias tool v.2 and evidence quality using Grading of Recommendations, Assessment, Development and Evaluation (GRADE).

**RESULTS:**

We included 7 trials (*n* = 687). The intervention was pulmonary vein isolation in 6 trials and ganglion plexi ablation in 1. Patients who received prophylactic ablation were less likely to have early postoperative atrial fibrillation (21% vs 37%, risk ratio [RR] 0.5, 95% confidence interval 0.3–0.8, *I*^2^ = 64%) and incident clinical atrial fibrillation at longest follow-up (range 6 months–2 years; 3% vs 10%, RR 0.3, 95% confidence interval 0.2–0.7, *I*^2^ = 0%). The quality of evidence was low.

**CONCLUSIONS:**

Prophylactic ablation during cardiac surgery may prevent atrial fibrillation in patients without a history of the arrhythmia. A definitive randomized trial is needed to confirm effects and safety.

## Graphical abstract

**Figure:**
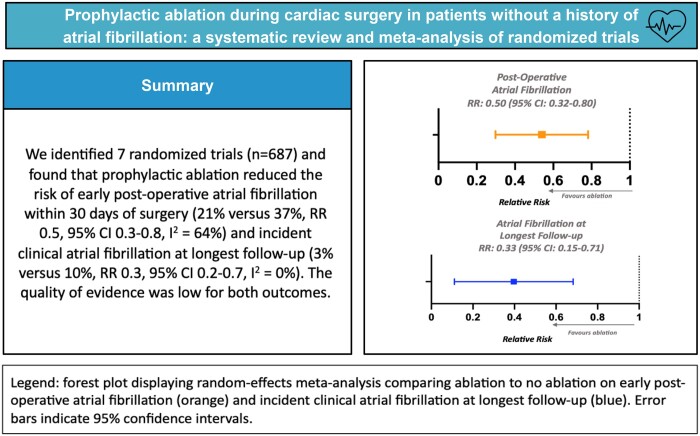

## INTRODUCTION

In North America and Europe, over a million adults undergo cardiac surgery annually, and this number is projected to increase [1–3]. Atrial fibrillation (AF) is the most common complication after cardiac surgery, occurring in up to 50% of patients undergoing such surgeries [4]. Early postoperative AF refers to new-onset AF (AF in patients without a history of the arrhythmia) developing within 4 weeks of cardiac surgery [4]. In the short-term, early postoperative AF is associated with an increased risk of short-term stroke and hospitalization [4, 5], and in the long-term, it is associated with clinical AF recurrence, stroke and death [4–8].

Although prophylactic therapies have been shown to reduce the incidence of early postoperative AF, their use is variable and the incidence of early postoperative AF remains high [4, 9, 10]. Ablation of arrhythmogenic atrial tissue, most commonly through isolation of the pulmonary veins, is a proven treatment for patients with established clinical AF [11–14]. Cardiac surgery offers direct access to the left atrium and pulmonary veins allowing concurrent preventative ablation at the time of operation [4, 12, 13, 15]. Whether or not prophylactic AF ablation during cardiac surgery is effective and safe is unknown.

This review aims to identify, appraise and synthesize evidence from randomized trials testing the efficacy of prophylactic atrial ablation in preventing AF among patients who undergo cardiac surgery.

## MATERIALS AND METHODS

We registered the study protocol with Prospero (CRD42023439701). The conduct and reporting of this study follows PRISMA guidelines [16]. Since this study uses publicly available documents as evidence, ethical approval was not required.

### Eligibility criteria

We included randomized trials enrolling patients 18 years or older undergoing any type of cardiac surgery, without a documented history of AF (including paroxysmal, persistent or permanent). The intervention of interest was surgical atrial ablation, including any lesion set or energy source, performed during cardiac surgery. The comparison group was patients who had cardiac surgery without ablation. We excluded trials in which catheter ablation occurred after cardiac surgery and those in which the ablation was a standalone procedure.

The primary outcome was new-onset early postoperative AF, defined as AF or atrial flutter of any duration within the first 30 days following surgery. Secondary outcomes were incident clinical AF at longest follow-up (minimum of 6 months), antiarrhythmic drug use (defined as use of vernakalant or class I or III agents at hospital discharge or in the first 30 postoperative days), oral anticoagulant use (at hospital discharge or in the first 30 postoperative days), thromboembolic events (including stroke or systemic arterial embolism), length of index hospital stay, length of index intensive care unit stay, surgery duration, cross-clamp time, cardiopulmonary bypass time, major postoperative bleeding and reoperation for bleeding. *Post-hoc* outcomes were mortality at longest follow-up, new permanent pacemaker implantation and left atrial flutter.

### Search methods

We searched CENTRAL, MEDLINE and Embase from inception to August 2024. The search strategy included subject headings and keywords pertaining to AF, ablation and cardiac surgery (Supplementary Material, Appendices 1–3). We used validated search filters to restrict our search strategy to randomized trials [17]. We did not place restraints based on language or publication status. For studies not in English, we used a translation module. An information specialist (J.Y.) reviewed and validated this search strategy.

### Trial selection process and data extraction

Trial selection for inclusion occurred in 2 phases: title and abstract screening, and full text review. Pairs of independent reviewers performed reviews using Covidence (Melbourne, Australia). We resolved disagreements through discussion with the senior review author. Two reviewers independently and in duplicate extracted data on the trial design, patient characteristics, interventions and ablation lesions, comparators and outcomes of the final included studies.

### Risk of bias assessment

We used the Cochrane Collaboration’s risk of bias tool v.2 to evaluate risk of bias in each trial [18]. Independently and in duplicate, 2 reviewers evaluated risk of bias as ‘low risk’, ‘some concerns’ or ‘high risk’ based on 5 domains: randomization process, deviations from intended interventions, missing outcome data, bias in measurement of the outcome and selection of the reported results. We labelled the overall risk of bias for each trial as ‘low risk’ if all domains were rated as ‘low risk’, ‘some concerns’ if at least 1 domain was rated as ‘some concerns’ with no domains rated as ‘high risk’ and ‘high risk’ if 1 or more domains were rated as ‘high risk’.

We generated rules *a priori* for evaluating risk of bias. We judged trials with unjustified post-randomization exclusions as ‘high risk’ of bias arising from deviations from intended interventions. When the trial did not provide a CONSORT diagram (or text necessary to generate one), we rated risk of bias arising from deviations from the intended interventions and missing outcome data as at least ‘some concerns’. For open-label trials, we assumed all parties, including the care team, the outcome assessors and patients, had knowledge of the group to which participants were assigned. If the care team was not blinded to treatment allocation, and there was no protocol for antiarrhythmic drug use, we judged risk of bias arising from deviations from intended interventions as ‘high risk’ for ‘antiarrhythmic use’ and as ‘some concerns’ for early postoperative AF, incident clinical AF at longest follow-up, stroke and/or systemic thromboembolism, length of hospital stay and length of intensive care unit stay. If the care team was not blinded to treatment allocation, and there was no protocol for anticoagulant use, we judged risk of bias arising from deviations from intended interventions as ‘high risk’ for anticoagulant use and as at least ‘some concerns for’ stroke and/or systemic thromboembolism. If there was no protocol for rhythm monitoring at a specific time point, either postoperatively or at follow-up, then we rated bias in measurement of the outcome as ‘some concerns’ for the outcomes of early postoperative AF or incident clinical AF at longest follow-up. We judged this as ‘high risk’ if there were obvious differences in the number or method of measurements between groups.

### Measures of association with treatment

We pooled data using dataparty.ca, following the intention-to-treat principle. For trials with multiple arms, we combined groups based on whether participants received ablation. For dichotomous or categorical data, we used the number of events in each arm to summarize across groups using the Mantel–Haenszel method and reported risk ratio with 95% confidence interval (CI). For continuous outcomes, we used the inverse variance method using the means and standard deviations reported in each included trial to report weighted mean difference with 95% CI. We pooled data using the DerSimonian–Laird method in random effects models and presented data using forest plots. We expected heterogeneity between trials; therefore, we chose to use random effects models. When trials presented medians and interquartile ranges, we transformed data to means and standard deviations in order to meta-analyse the results [19]. We assessed heterogeneity using the χ^2^ test for homogeneity, *I*^2^ test for inconsistency and by conducting subgroup analyses.

### Subgroup analysis

We pre-specified subgroup analyses for early postoperative AF and incident clinical AF at longest follow-up. Subgroup analyses were based on risk of bias in included trials, type of cardiac surgery [isolated coronary artery bypass graft (CABG) versus other cardiac surgeries], ablation lesion set and type of rhythm monitoring. After data collection, we conducted further *post-hoc* subgroup analyses based on protocol for antiarrhythmic drug use, protocol for anticoagulant drug use and use of amiodarone in the comparator group. To estimate the effect of individual studies on the pooled effect-size estimate, we conducted leave-one-out meta-analyses for the early postoperative AF and incident clinical AF at longest follow-up.

### Assessment of quality of evidence

We used the Grading of Recommendations, Assessment, Development and Evaluation (GRADE) approach to assess the certainty of the evidence for each outcome [20]. GRADE evaluates the certainty of evidence based on overall risk of bias, heterogeneity of the data, effect size estimate precision, directness of the evidence and risk of publication bias. According to GRADE, data from randomized trials begin as high-quality evidence; this rating can decrease if concerns arise. Since we identified fewer than 10 studies for inclusion, we did not assess publication bias using a funnel plot.

## RESULTS

### Screening

**Figure 1:**
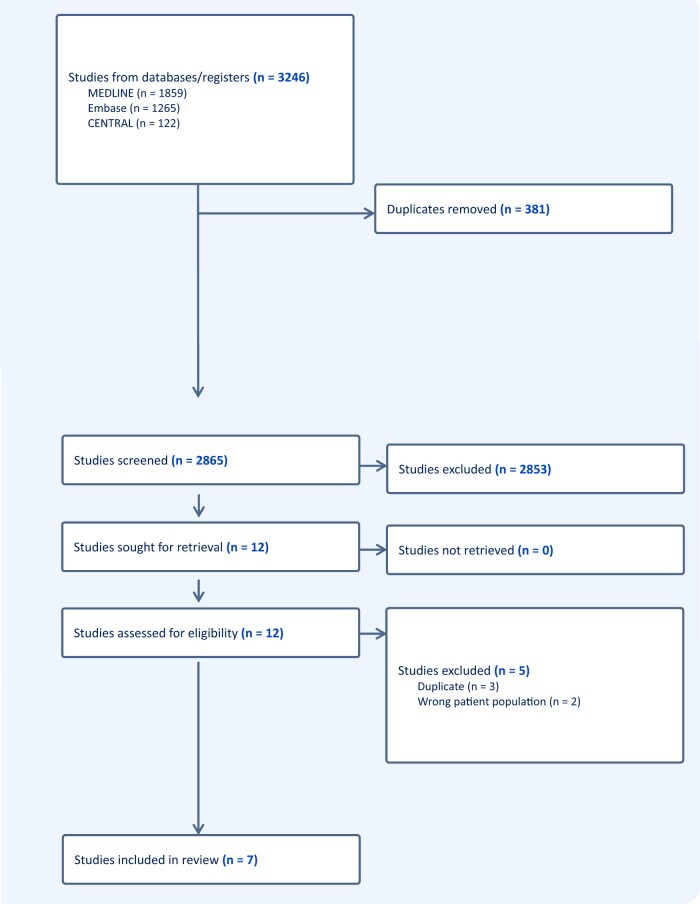
PRISMA flow diagram: overview of study selection process.

Our search strategy identified 2865 unique title and abstracts for screening; we reviewed 12 full texts for eligibility, and 7 randomized trials met inclusion criteria (Fig. 1).

### Included trials

The 7 randomized trials collectively included 687 patients (mean age: 66.0 ± 6.8 years, 15.8% women) undergoing cardiac surgery (Table 1, with further details in Supplementary Material, Appendices 4 and 5) [21–27]. Six trials included patients undergoing isolated CABG surgery, while 1 other included patients undergoing either CABG, aortic valve replacement or combined CABG–aortic valve replacement surgery [27]. Six trials tested pulmonary vein isolation, while the other examined ablation of autonomic ganglion plexi [21]. Radiofrequency was the energy source in all trials. One trial was published only as an abstract [24]. Two trials did not use any antiarrhythmic drug prophylaxis in the control arm, 3 used beta-blockers and 2 used amiodarone. Five trials assessed early postoperative AF by continuously monitoring patients’ surface electrocardiogram in-hospital during the postoperative period, 1 study used an implanted monitor and 1 study did not describe their method of rhythm monitoring. Four trials had protocols for antiarrhythmic drug use, and 1 ensured a protocol for oral anticoagulant use. Five trials assessed rhythm at follow-up; 2 used continuous monitoring, 1 used an implanted monitor, 1 reviewed medical records and interviewed patients, and 1 did not describe their method of follow-up.

**Table 1:** Summary and characteristics of included trials

Study ID	Surgery (*n*)	Males, *n* (%)	Age (years), mean ± SD	Treatment group(s)	Comparison group(s)	Co-interventions	Early postoperative atrial fibrillation definition	Protocols for anticoagulant, antiarrhythmic	Inpatient rhythm assessment	Outpatient follow-up length	Method of rhythm assessment at follow-up
Al-Atassi *et al.* 2014	CABG (47)	45 (95.7%)	60.8 ± 10.3	Autonomic ganglion plexus ablation by radiofrequency	No prophylaxis	None prespecified	Detectable atrial fibrillation before discharge >5 min in length, or requiring intervention to control rate, relive symptoms or restore haemodynamics or postoperative atrial flutter	Anticoagulant: N	Continuous monitoring	None	No follow-up
								Antiarrhythmic: N			
Kiaii *et al.* 2015	CABG (175)	156 (89.1%)	69.0 ± 7.9	Pulmonary vein isolation byradiofrequency and ß-blocker	ß-blocker	None prespecified	Continuous AF detected on telemetry/ECG for ≥5 min requiring treatment or asymptomatic atrial fibrillation lasting for >30 min while patients were in hospital	Anticoagulant: N	Continuous monitoring	6 months	ECG and 48-h Holter monitor
								Antiarrhythmic: N			
Lednev *et al.* 2017	CABG (117)	NR	NR	Pulmonary vein isolation by radiofrequency	No prophylaxis	None prespecified	No definition	Anticoagulant: N	Not reported	1 year	Not reported
					Amiodarone			Antiarrhythmic: Y			
Revishvili *et al.* 2023	CABG (175)	144 (82.2%)	61.3 ± 6.3	Pulmonary vein isolation by radiofrequency	No prophylaxis	None prespecified	No definition	Anticoagulant: N Antiarrhythmic: Y	Continuous monitoring	1 year	48-h Holter
				Pulmonary vein isolation by radiofrequency and amiodarone	Amiodarone						
Revishvili *et al.* 2020	CABG (63)	54 (85.7%)	61.2 ± 6.6	Pulmonary vein isolation by radiofrequency	No prophylaxis	After surgery, resumed beta-blocker therapy for patients who were on beta-blockers pre-operatively	Episodes longer than 5 min considered significant	Anticoagulant: N	Continuous monitoring for 48 h then discrete monitoring	None	No follow-up
								Antiarrhythmic: Y			
Teijeira *et al.* 2014	CABG (50)	42 (84%)	71.6 ± 4.6	Pulmonary vein isolation by radiofrequency with exclusion of the LAA	No prophylaxis	None prespecified	Episodes lasting 2 min or longer	Anticoagulant: N	Implanted monitor	2 years	Implanted monitor
								Antiarrhythmic: N			
Willekes *et al.* 2023	CABG (50)	39 (65%)	75 ± 4	PVI by RFA with LAA amputation	No prophylaxis	All patients with CAD received beta-blockers	Irregular heart rhythm without P waves >30 s; postoperative atrial flutter	Anticoagulant: Y	Continuous monitoring	1 year	Electronic medical record review and phone interview
	AVR (3)							Antiarrhythmic: Y			
	AVR/+ CABG (7)										

AGP: autonomic ganglion plexus; AVR: aortic valve replacement; CAD: coronary artery disease; CABG: coronary artery bypass graft; ECG: electrocardiogram; LAA: left atrial appendage; PVI: pulmonary vein isolation; RFA: radiofrequency ablation.

### Risk of bias

For the primary outcome of early postoperative AF, we rated 3 trials as ‘high risk’ of bias due to post-randomization exclusions and the absence of reporting on outcomes specified in the protocol. We rated the other 4 trials as having ‘some concerns’ due to factors such as lack of blinding, lack of protocol for arrhythmic use, unreported method for measuring early postoperative AF and unreported randomization method. Details of risk of bias judgements appear in Supplementary Material, Appendices 4 and 6.

### Early postoperative atrial fibrillation within 30 days of surgery

**Figure 2:**
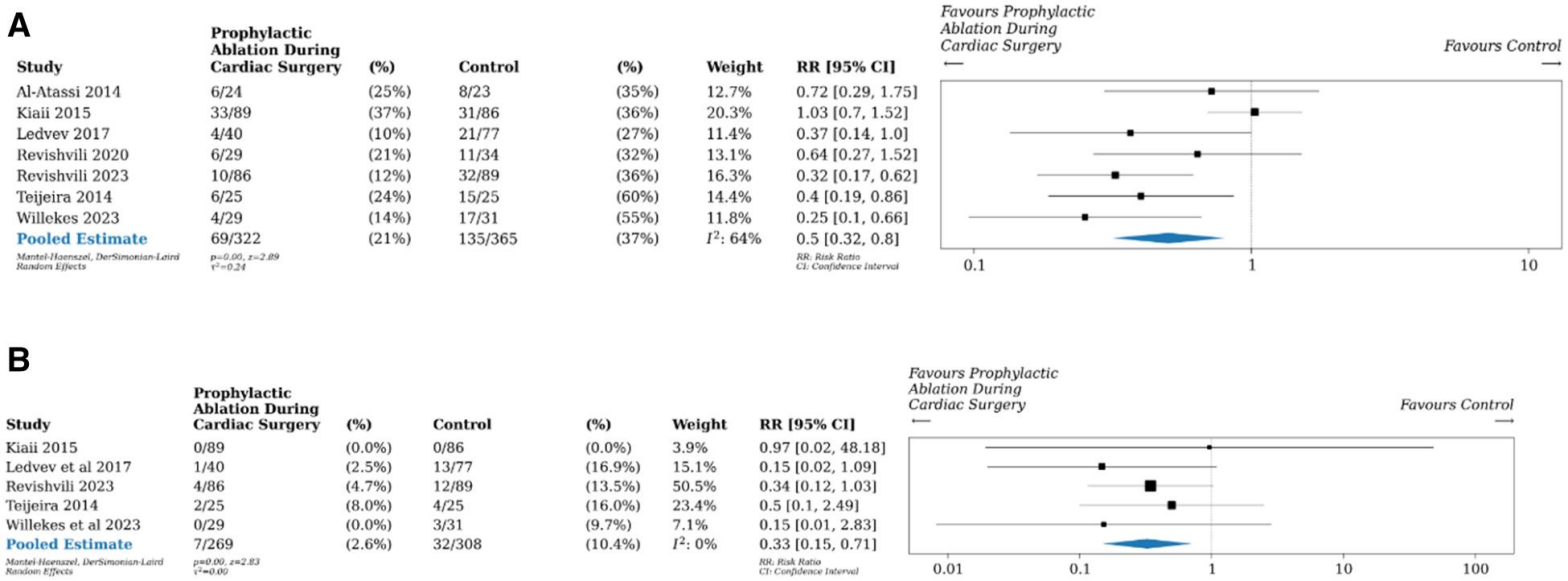
Relative risk of early postoperative AF within 30 days and incident clinical AF at longest follow-up: (**A**) Forest plot displaying random-effects meta-analysis comparing ablation to no ablation on early postoperative AF within 30 days. Error bars indicate 95% confidence intervals. (**B**) Forest plot displaying random-effects meta-analysis comparing ablation to no ablation on incident clinical AF at longest follow-up. Error bars indicate 95% confidence intervals.

All 7 trials (687 patients, 204 events) reported on early postoperative AF. In pooled analyses, early postoperative AF was detected in a significantly lower proportion of patients who received prophylactic intraoperative ablation (21% vs 37%, RR 0.5, 95% CI 0.3–0.8, *I*^2^ = 64%, Fig. 2A) compared to those who did not. We were unable to conduct subgroup analyses based on type of surgery as the 1 trial that included non-CABG patients did not report outcomes for different types of surgery. In other subgroup analyses, neither risk of bias, ablation approaches, method of rhythm assessment, use of amiodarone in comparator group, protocol for antiarrhythmic use, protocol for anticoagulant use, nor LAAO as a co-intervention had a statistically significant impact on the outcome (Supplementary Material, Appendix 7). The results of the leave-one-out meta-analyses were similar to the meta-analysis including all trials (Supplementary Material, Appendix 8). We judged the quality of evidence for this outcome based on the GRADE framework to be low due to risk of bias and inconsistency (Supplementary Material, Appendix 9).

### Incident clinical atrial fibrillation at longest follow-up

Five trials (577 patients, 39 events) followed participants after hospital discharge and reported on incident clinical AF at long-term follow-up. One trial followed patients for 6 months, 3 trials followed patients for 1 year and 1 trial followed patients for 2 years (mean: 13 months, median: 12 months; Supplementary Material, Appendix 10). Patients receiving prophylactic intraoperative ablation were less likely to have incident clinical AF at longest follow-up (3% vs 10%, RR 0.3, 95% CI 0.2–0.7, *I*^2^ = 0%, Fig. 2B). Subgroup analyses demonstrated that neither risk of bias, ablation approach, method of rhythm assessment, use of amiodarone in comparator group, protocol for antiarrhythmic use, protocol for anticoagulant use, nor LAAO as a co-intervention had a statistically significant impact on the outcome (Supplementary Material, Appendix 7). The results of the leave-one-out meta-analyses were similar to the meta-analysis including all trials (Supplementary Material, Appendix 8). We judged the quality of evidence for this outcome to be low due to risk of bias and indirectness (variation in outcome measurement) (Supplementary Material, Appendix 9).

### Antiarrhythmic and anticoagulant drug use

Four trials (332 patients, 91 events) reported on antiarrhythmic drug use after surgery (Supplementary Material, Appendices 11 and 12). Patients receiving prophylactic intraoperative ablation were less likely to receive rhythm control with antiarrhythmic drugs compared to the control group (20% vs 35%, RR 0.5, 95% CI 0.2–1.1, *I*^2^ = 63%, Supplementary Material, Appendix 11). Four trials (332 patients, 44 events) reported on anticoagulant use after surgery. Ablated patients were less likely to be prescribed anticoagulants (8% vs 18%, RR 0.4, 95% CI 0.2–1.0, *I*^2^ = 21%, Supplementary Material, Appendix 11).

Four studies reported protocols for antiarrhythmic use, while 1 had a protocol for anticoagulant use. Subgroup analyses demonstrated that protocol for antiarrhythmic use or anticoagulant use did not have a statistically significant impact on their respective outcomes (Supplementary Material, Appendix 7). We judged the quality of evidence based on the GRADE framework to be very low for antiarrhythmic use due risk of bias, imprecision and inconsistency and low for anticoagulant use due to risk of bias and imprecision (Supplementary Material, Appendix 9).

### Length of stay

Six trials reported on length of hospital stay (508 patients; Supplementary Material, Appendix 11). We found no significant difference in length of hospital stay between the 2 groups (9.3 days vs 9.9 days, mean difference <0.1 days, 95% CI –1.3 to 1.3, *I*^2^ = 78%, Supplementary Material, Appendix 11). Three trials (287 patients) reported on length of intensive care unit stay. Intensive care unit stay was similar between the 2 groups (50 h vs 43 h, mean difference –2.1 h, 95% CI –16.2–12.1, *I*^2^ = 47%, Supplementary Material, Appendix 11). We judged the quality of evidence for length of hospital stay as very low due to risk of bias, inconsistency, imprecision and data not being normally distributed, and the quality of evidence for length of intensive care unit stay as very low due to risk of bias, imprecision and data not being normally distributed (Supplementary Material, Appendix 9). Subgroup analyses demonstrated that neither use of amiodarone in comparator group, protocol for antiarrhythmic use, nor protocol for anticoagulant use had a statistically significant effect on length of hospital stay. Protocol for antiarrhythmic use did not have a statistically significant effect on length of intensive care unit stay (Supplementary Material, Appendix 7).

### Surgery duration

Four trials (405 patients) reported on surgery duration, 5 trials (465 patients) reported on cardiopulmonary bypass time and 4 trials (326 patients) reported on cross-clamp time (Supplementary Material, Appendix 11). Surgery duration (239 vs 213 min, mean difference 16 min, 95% CI 1–31, *I*^2^ = 70%, Supplementary Material, Appendix 11) and cardiopulmonary bypass time (94 vs 67 min, mean difference 13 min, 95% CI 7–18, *I*^2^ = 43%, Supplementary Material, Appendix 11) were significantly longer in the ablation group. Cross-clamp time was not significantly different (64 vs 59 min, mean difference 5 min, 95% CI –2–13, *I*^2^ = 76%, Supplementary Material, Appendix 11). We judged the quality of evidence for surgery duration to be very low due to risk of bias, imprecision and inconsistency; we judged the quality of evidence for cross-clamp time to be very low due to risk of bias, imprecision and inconsistency, and cardiopulmonary bypass time to be moderate due to risk of bias (Supplementary Material, Appendix 9).

### Complications

Four trials reported on reoperation for bleeding (402 patients, 10 events; Supplementary Material, Appendix 11). We found no difference in the likelihood of requiring reoperation for bleeding with prophylactic ablation (3% vs 2%, RR 1.6, 95% CI 0.5–5.3, *I*^2^ = 0%, Supplementary Material, Appendix 11). We judged the quality of evidence for this outcome to be low due to risk of bias and imprecision (Supplementary Material, Appendix 9). No trials reported on major postoperative bleeding. Only 1 trial (50 patients, no events) reported on left-atrial flutter; therefore, results were not meta-analysed [26].

### Stroke, mortality and new permanent pacemaker implantation

Four trials (332 patients, 3 events, with follow-up unknown, 6 months, 24 months, unknown) reported on stroke (Supplementary Material, Appendix 11). We found no evidence of difference in the risk of stroke with ablation (1% vs 1%, RR 0.7, 95% CI 0.1–3.9, *I*^2^ = 0%, Supplementary Material, Appendix 11). We judged the quality of evidence to be very low for this outcome due to risk of bias and very serious imprecision (Supplementary Material, Appendix 9). Subgroup analysis demonstrated that protocol for anticoagulant use did not have a statistically significant effect on the risk of stroke (Supplementary Material, Appendix 7). Six trials (640 patients, 6 events, with follow-up unknown, in-hospital, 6 months, 1 year (2), and 2 years; mean: 11 months, median: 12 months) reported on mortality. The risk of mortality with ablation was not different compared with patients in the control group (1% vs 1%, RR 0.8, 95% CI 0.2–2.8, *I*^2^ = 0%). One trial (60 patients, no events) reported on new permanent pacemaker implantation; therefore, results were not meta-analysed [27].

## DISCUSSION

This systematic review and meta-analysis of randomized trials found that prophylactic ablation during cardiac surgery may prevent early postoperative AF within 30 days of surgery and incident clinical AF at longest follow-up. Moreover, patients who received ablation were less likely to receive antiarrhythmic drugs or oral anticoagulation. We observed no difference in stroke or bleeding. However, the existing evidence base has important limitations related to the design and conduct of the included randomized trials.

Prevention of early postoperative AF has long been a major focus of cardiac surgery research; many pharmacologic therapies and techniques have been studied and several have been shown to be effective (e.g. amiodarone, beta-blockers, sotalol, magnesium, atrial pacing and left/posterior pericardiotomy) [9, 28]. Despite these advances, the incidence of early postoperative AF remains high [10]. Several mechanisms may drive early postoperative AF, including atrial substrate, pericardial effusion and inflammation, adipose tissue metabolism, changes in ion channels and gap junctions, the autonomic nervous system and pulmonary vein triggers [29]. Pulmonary vein isolation with catheter ablation only affects 1 mechanism (i.e. pulmonary vein triggers), while many other proarrhythmic mechanisms for the development of early postoperative AF exist transiently after surgery and then normalize. This means the efficacy of prophylactic intraoperative ablation for early postoperative AF prevention in patients undergoing cardiac surgery could be less pronounced compared to its efficacy for prevention of clinical AF over long-term follow-up. This is consistent with the trend we observed with prophylactic intraoperative ablation in this study, showing an estimated 50% relative risk reduction in early postoperative AF and a 66% reduction in incident clinical AF over the long-term period.

This review provides encouraging evidence for prophylactic ablation at the time of cardiac surgery. However, the included studies have important limitations preventing the implementation of their findings into clinical practice. The number of patients in each study is small, and the totality of randomized patients is <1000. While we intended to include any type of cardiac surgery, more than 98.5% of all patients underwent isolated CABG—thus our results may not be generalizable to other types of surgery. We cannot be sure that all trials made rigorous efforts to exclude patents with a preoperative history of AF. The open-label nature of the trials also leads to the possibility of differential management between study groups. Future trials should consider blinding—the Left Atrial Appendage Occlusion Study III (LAAOS III) showed that blinding of care team members other than those in the operating theatre is feasible [30]. Objective criteria for initiation and discontinuation of antiarrhythmic drugs and anticoagulants may act as an additional safeguard against bias. To be credible, monitoring for AF over long-term follow-up needs to proceed in a structured fashion. In this review, the rate of incident clinical AF at longest follow-up was 3% in ablated patients and 10% in non-ablated patients. The AF incidence in the control arm is markedly lower than has been seen in population-based studies in the non-surgical setting; in these studies, an AF incidence of at least 20% has consistently been seen in the first 2 years after implantation of an implanted cardiac monitor [31–34]. In a systematic review of patients with early postoperative AF after cardiac surgery who received an implanted cardiac monitor, we found that the incidence of AF at 1 year post-surgery was roughly 1 in 3. [35] Use of an implanted cardiac monitor would increase event capture and ensure all study patients are monitored in the same way. Because included studies reported only the crude proportions of patients with an outcome at any given time point and not annualized rates, we are unable to calculate or pool incidence rate ratios. Finally, an important consideration that was not assessed by studies in this review was the effect of ablation on AF-related long-term adverse outcomes. Existing studies have shown mixed effects on the association of early postoperative AF and long-term outcomes including stroke, heart failure and cardiovascular death [5, 8]. AF is an important mediator of these outcomes. An appropriately designed study must consider these and other AF-related outcomes over long-term follow-up [36, 37].

## CONCLUSION

Low-quality evidence suggests that in patients without a history of AF, prophylactic ablation concomitant to cardiac surgery may decrease both early postoperative AF within 30 days and incident clinical AF in longer follow-up. Existing randomized trials are small, open-label, assess AF using variable methods and did not track long-term clinical outcomes. A large, well-designed randomized trial that addresses these limitations is needed.

## Supplementary Material

ivae195_Supplementary_Data

## Data Availability

All relevant data are within the manuscript and its Supporting Information files. The data are derived from published manuscripts.

## Notes

Presented at the American College of Cardiology Annual Scientific Session 2024, Atlanta, GA, USA, 6 April 2024.

## ABBREVIATIONS

AF: Atrial fibrillation
CABG: Coronary artery bypass graft
CI: Confidence interval
GRADE: Grading of Recommendations, Assessment, Development, and Evaluations
LAAOS II: Left Atrial Appendage Occlusion Study III
