# Supplementary material for: Prophylactic ablation during cardiac surgery in patients without atrial fibrillation: a systematic review and meta-analysis of randomized trials
Source: Interdiscip Cardiovasc Thorac Surg. 2024 Nov 26;39(6):ivae195. doi: 10.1093/icvts/ivae195 (PMC11661978; doi:10.1093/icvts/ivae195)

**Appendix 1. MEDLINE Search Strategy**

**Appendix 2. Embase Search Strategy**

**Appendix 3. Cochrane CENTRAL Search Strategy**

**Appendix 4. Characteristics of Included Trials**

**Appendix 5. Outcomes Reported in Included Trials**

**Appendix 6. Sources of Risk of Bias in Included Trials**

**Appendix 7. Subgroup Analyses**

**Appendix 1: MEDLINE Search Strategy**

Database(s): Ovid MEDLINE(R) ALL 1946 to August 26, 2024 Search Strategy:

# Searches

1 Atrial Fibrillation/ (75653)

2 atrial fib*.mp. (114755)

3 afib.mp. (552)

4 atrium fibrillation*.mp. (13)

5 a fib.mp. (291)

6 POAF.mp. (930)

7 atrium fibrillation*.mp. (13)

8 AF.mp. (73077)

9 1 or 2 or 3 or 4 or 5 or 6 or 7 or 8 (147960)

10 exp Cardiac Surgical Procedures/ (252011)

11 exp Heart/su (68109

12 exp Heart Diseases/su (207959

13 ((cardiac or cardio* or cardial or heart*) adj2 (surger* or operat*)).mp. (93569)

14 coronary artery bypass graft*.mp. (39457)

15 CABG.mp. (22436)

16 valve replacement*.mp. (49665)

17 Thoracic Surgery/ (13991)

18 10 or 11 or 12 or 13 or 14 or 15 or 16 or 17 (438774)

19 Catheter Ablation/ (40965)

20 Radiofrequency Ablation/ (3206)

21 ablat*.mp. (159866)

22 19 or 20 or 21 (159866)

23 ((randomized controlled trial or controlled clinical trial).pt. or randomized.ab. or randomised.ab. or placebo.ab. or drug therapy.fs. or randomly.ab. or trial.ab. or groups.ab.) not (exp animals/ not humans.sh.) (5316711)

24 exp Postoperative Complications/ (632267)

25 exp Postoperative Care/ (61026)

26 ae.fs. (2070106)

27 mo.fs. (640817)

28 exp Prophylactic Surgical Procedures/ (755)

29 postoperative.mp. (975429)

30 post operative.mp. (83419)

31 prevent*.mp. (2970942)

32 prophyla*.mp. (217750)

33 24 or 25 or 26 or 27 or 28 or 29 or 30 or 31 or 32 (5911293)

34 9 and 18 and 22 and 23 and 33 (2037)

**Appendix 2: Embase Search Strategy**

Database(s): Embase <1974 to 2024 August 26 >

Search Strategy:

--------------

1 exp atrial fibrillation/ (144844)

2 atrial fibrillation*.mp. (222447)

3 atrium fibrillation*.mp. (86628)

4 auricular fibrillation*.mp. (448)

5 afib.mp. (2082)

6 a fib.mp. (840)

7 POAF.mp. (1497)

8 AF.mp. (119205)

9 or/1-8 (288248)

10 exp heart surgery/ (471380)

11 ((cardiac or cardio* or cardial or heart*) adj2 (surger* or operat*)).mp. (193021)

12 coronary artery bypass graft*.mp. (105849)

13 CABG.mp. (41472)

14 valve replacement*.mp. (91171)

15 or/10-14 (541491)

16 exp catheter ablation/ (46269)

17 exp ablation device/ (14181)

18 exp ablation therapy/ (73481)

19 ablat*.mp. (259148)

20 16 or 17 or 18 or 19 (260393)

21 (Randomized controlled trial/ or Controlled clinical study/ or random$.ti,ab. or randomization/ or intermethod comparison/ or placebo.ti,ab. or (compare or compared or comparison).ti. or ((evaluated or evaluate or evaluating or assessed or assess) and (compare or compared or comparing or comparison)).ab. or (open adj label).ti,ab. or ((double or single or doubly or singly) adj (blind or blinded or blindly)).ti,ab. or double blind procedure/ or parallel group$1.ti,ab. or (crossover or cross over).ti,ab. or ((assign$ or match or matched or allocation) adj5 (alternate or group$1 or intervention$1 or patient$1 or subject$1 or participant$1)).ti,ab. or (assigned or allocated).ti,ab. or (controlled adj7 (study or design or trial)).ti,ab. or (volunteer or volunteers).ti,ab. or human experiment/ or trial.ti.) not (((random$ adj sampl$ adj7 ("cross section$" or questionnaire$1 or survey$ or database$1)).ti,ab. not (comparative study/ or controlled study/ or randomi?ed controlled.ti,ab. or randomly assigned.ti,ab.)) or (Cross-sectional study/ not (randomized controlled trial/ or controlled clinical study/ or controlled study/ or randomi?ed controlled.ti,ab. or control group$1.ti,ab.)) or (((case adj control$) and random$) not randomi?ed controlled).ti,ab. or (Systematic review not (trial or study)).ti. or (nonrandom$ not random$).ti,ab. or "Random field$".ti,ab. or (random cluster adj3 sampl$).ti,ab. or ((review.ab. and review.pt.) not trial.ti.) or ("we searched".ab. and (review.ti. or review.pt.)) or "update review".ab. or (databases adj4 searched).ab. or ((rat or rats or mouse or mice or swine or porcine or murine or sheep or lambs or pigs or piglets or rabbit or rabbits or cat or cats or dog or dogs or cattle or bovine or monkey or monkeys or trout or marmoset$1).ti. and animal experiment/) or (Animal experiment/ not (human experiment/ or human/))) (5914714)

22 exp prevention/ (1841369)

23 prophyl*.mp. (398112)

24 prevent*.mp. (3523786)

25 exp postoperative complication/ (874269)

26 22 or 23 or 24 or 25 (5172583)

27 9 and 20 and 21 and 26 (1374)

***************************

**Appendix 3: Cochrane CENTRAL Search Strategy**

Date run: 7/3/23, 8:22 PM

Search Strategy:

--------------

1 Atrial Fibrillation/ (7337)

2 atrial fib*.mp. (16822)

3 afib.mp. (116)

4 atrium fibrillation*.mp. (801)

5 a fib.mp. (39)

6 POAF.mp. (253)

7 atrium fibrillation*.mp. (801)

8 AF.mp. (10143)

9 1 or 2 or 3 or 4 or 5 or 6 or 7 or 8 (19678)

10 exp Cardiac Surgical Procedures/ (17316)

11 exp Heart/su (121)

12 exp Heart Diseases/su (133)

13 ((cardiac or cardio* or cardial or heart*) adj2 (surger* or operat*)).mp. (18150)

14 coronary artery bypass graft*.mp. (10294)

15 CABG.mp. (6923)

16 valve replacement*.mp. (3373)

17 Thoracic Surgery/ (247)

18 10 or 11 or 12 or 13 or 14 or 15 or 16 or 17 (36763)

19 Catheter Ablation/ (2311)

20 Radiofrequency Ablation/ (207)

21 ablat*.mp. (13092)

22 19 or 20 or 21 (13092)

23 ((randomized controlled trial or controlled clinical trial).pt. or randomized.ab. or randomised.ab. or placebo.ab. or drug therapy.fs. or randomly.ab. or trial.ab. or groups.ab.) not (exp animals/ not humans.sh.) (1443546)

24 exp Postoperative Complications/ (55330)

25 exp Postoperative Care/ (5371)

26 ae.fs. (182223)

27 mo.fs. (37514)

28 exp Prophylactic Surgical Procedures/ (32)

29 postoperative.mp. (152555)

30 post operative.mp. (29118)

31 prevent*.mp. (294567)

32 prophyla*.mp. (45128)

33 24 or 25 or 26 or 27 or 28 or 29 or 30 or 31 or 32 (574196)

34 9 and 18 and 22 and 23 and 33 (134)

*************************

**Appendix 4 Characteristics of Included Trials:**

**Al-Atassi 2014:**

| Methods | Single-centre randomized controlled trial at a hospital in Ontario, Canada | |
| --- | --- | --- |
| Inclusion Criteria | - Adult patients - Scheduled for isolated CABG surgery | |
| Exclusion Criteria | - Previous history of AF, ablation, brady-tachyarrhythmias, sick sinus syndrome, valvular disease, secundum atrial septal defect requiring intervention - Planned for off pump or minimally invasive coronary revascularization, redo or emergency surgery - Left main disease or left ventricular ejection fraction < 30% - Language barriers or inability to give consent | |
| Interventions | Mapping and ablation of autonomic ganglion plexus  versus  No prophylaxis | |
| Population Characteristics | Ablation:   - Age (mean, SD): 60.9 ± 10.0 years - Male (%, n): 95.8% (23/24) - Asthma/COPD (%, n): 20.8% (5/24) - BMI (mean, SD): 30.6 ± 4.7 - Hypertension (%, n): 58.3% (14/24) - Diabetes (%, n): 25.0% (6/24) - COPD: (%, n): 20.8% (5/24) - Preoperative beta-blockers (%, n): 70.8% (17/24) - History of myocardial infarction (%, n): 33.3% (8/24) - History of peripheral vascular disease (%, n): 8.3% (2/24)   Control:   - Age (mean, SD): 60.6 ± 10.6 years - Male (n, %): 95.7% (22/23) - Asthma/COPD (%, n): 8.7% (2/23) - BMI (mean, SD): 30.5 ± 5.4 - Hypertension (%, n): 73.9% (17/23) - Diabetes (%, n): 39.1% (9/23) - COPD (%, n): 8.7% (2/23) - Preoperative beta-blockers (%, n): 69.6% (16/23) - History of myocardial infarction (%, n): 26.1% (6/23) - History of peripheral vascular disease (%, n): 4.3% (1/23) | |
| Outcomes Reported in Manuscript and Definitions | - Early post-operative AF (post-operative atrial fibrillation; detectable atrial fibrillation before discharge > 5 minutes in length, or requiring intervention to control rate, relive symptoms, or restore hemodynamics or post-operative atrial flutter) - Antiarrhythmic use (post-operative amiodarone) - Anticoagulant use (post-operative warfarin) - Stroke (stroke or transient ischemic attack in hospital) - Length of hospital stay | |
| Protocol for Antiarrhythmic Use | No protocol | |
| Protocol for Anticoagulant Use | No protocol | |
| Outcomes Clarified by Contacting Authors | Authors contacted regarding surgery duration and length of hospital say. No data available for surgery duration, clarified length of hospital stay. | |
| Potential Conflicts | Funding source: unrestricted grant from the University of Ottawa Heart Institute and equipment in kind from Medtronic Inc  Declarations of interest: No disclosures | |
| Notes | N/A | |
| ***Risk of bias*** | | |
| *Bias domain* | *Authors’ judgement* | *Support for judgement* |
| Domain 1: Risk of bias arising from the randomization process | Low risk | Patients were assigned according to a computer-generated random number list |
| Domain 2: Risk of bias due to deviations from the intended interventions (effect of assignment to intervention)  *Early post-operative AF, length of hospital stay, stroke* | Some concerns | Unknown whether care team was blinded and no protocol for antiarrhythmic and anticoagulant use |
| Domain 2: Risk of bias due to deviations from the intended interventions (effect of adhering to intervention)  *Antiarrhythmic use, anticoagulant use* | High risk | Unknown whether care team was blinded and no protocol for anticoagulant and antiarrhythmic use |
| Domain 2: Risk of bias due to deviations from the intended interventions (effect of adhering to intervention)  *Early post-operative AF, length of hospital stay, stroke* | Some concerns | Unknown whether care team was blinded and no protocol for anticoagulant and antiarrhythmic use |
| Domain 3: Missing outcome data  *All outcomes* | Low risk | All patients accounted for |
| Domain 4: Risk of bias in measurement of the outcome  *All outcomes* | Low risk | Objective outcomes and standard method of rhythm assessment |
| Domain 5: Risk of bias in selection of the reported result | Low risk | No protocol but standard outcomes |
| Overall risk of bias  *Antiarrhythmic use, anticoagulant use* | High risk | |
| Overall risk of bias  *Early post-operative AF, length of hospital stay, stroke* | Some concerns | |

**Kiaii 2015:**

| Methods | Single-centre randomized controlled trial in Ontario, Canada | |
| --- | --- | --- |
| Inclusion Criteria | - Adult patients with coronary artery disease - Scheduled for elective coronary artery bypass graft (CABG) surgery with cardiopulmonary bypass - Meeting one of the following criteria: age ≤ 65 years, ejection fraction ≥ 40%, hypertension, or diabetes | |
| Exclusion Criteria | - Previous history of AF, antiarrhythmic use - Concomitant valve procedure or off-pump CABG surgery - Enrolled in another study - Inability to obtain consent - Contraindications to ß-blockers - Unable to attend follow-up visit | |
| Interventions | Radiofrequency ablation of pulmonary veins and post-operative ß-blocker  versus  Post-operative ß-blocker | |
| Population Characteristics | Ablation:   - Age (mean, SD): 69.6 ± 7.2 years - Male (%, n): 88.8% (79/89) - CHADS2 score (mean, SD): 1.3 ± 1.0 - Left atrium size (mean): 3.7 cm - BMI (mean, SD): 30.0 ± 4.8 - Hypertension (%, n): 82.0% (73/89) - Diabetes (%, n): 33.7% (30/89) - COPD (%, n): 11.2% (10/89) - Preoperative hemoglobin (mean, SD): 140.4 ± 16.0 g/L - History of myocardial infarction (%, n): 24.7% (22/89) - History of stroke (%, n): 9.0% (8/89) - History of peripheral vascular disease (%, n): 7.9% (8/89)   Control:   - Age (mean, SD): 68.3 ± 8.5 years - Male (%, n): 89.5% (77/86) - CHADS2 score (mean, SD): 1.3 ± 1.0 - Left atrium size (mean): 3.9 cm - BMI (mean, SD): 30.2 ± 5.6 - Hypertension (%, n): 74.4% (64/86) - Diabetes (%, n): 38.4% (33/86) - COPD (%, n): 9.3% (8/86) - Preoperative hemoglobin (mean, SD): 136.6 ± 14.2 g/L - History of myocardial infarction (%, n): 20.9% (18/86) - History of stroke: 12.8% (11/86) - History of peripheral vascular disease (%, n): 11.6% (10/86) | |
| Outcomes Reported in Manuscript and Definitions | - Early post-operative AF (continuous AF detected on telemetry/ECG for ≥ 5 minutes requiring treatment or asymptomatic atrial fibrillation lasting for > 30 minutes while patients were in hospital) - Incident clinical AF at longest follow-up (atrial fibrillation detected during 6 month follow-up; detected using ECGs and 48-hour Holter monitors from follow-up visit) - Antiarrhythmic use (post-operative amiodarone) - Anticoagulant use (post-operative warfarin) - Stroke (stroke during follow-up period; obtained from hospital records) - Length of hospital stay - Length of intensive care unit stay - Surgery duration - Cross-clamp time - Cardiopulmonary bypass time - Reoperations for bleeding - Mortality (6 months) | |
| Protocol for Antiarrhythmic Use | No protocol | |
| Protocol for Anticoagulant Use | No protocol | |
| Outcomes Clarified by Contacting Authors | None | |
| Potential Conflicts | Funding source: Internal research grant from Western University, and equipment in kind from Medtronic Inc  Declarations of interest: Three authors are consultants for Medtronic | |
| Notes | N/A | |
| ***Risk of bias*** | | |
| *Bias domain* | *Authors’ judgement* | *Support for judgement* |
| Domain 1: Risk of bias arising from the randomization process | Some concerns | No randomization method specified, however balanced baseline parameters |
| Domain 2: Risk of bias due to deviations from the intended interventions (effect of assignment to intervention) | High risk | 18/193 (9.3%) of participants were excluded from analysis post-randomization. Unknown whether care team was blinded. No protocol for anticoagulant, antiarrhythmic use. |
| Domain 2: Risk of bias due to deviations from the intended interventions (effect of adhering to intervention)  *Antiarrhythmic use, anticoagulant use* | High risk | Unknown whether care team was blinded. No protocol for anticoagulant, antiarrhythmic use. |
| Domain 2: Risk of bias due to deviations from the intended interventions (effect of adhering to intervention)  *Early post-operative AF, incident clinical AF at longest follow-up, stroke, length of hospital stay, length of intensive care unit stay, mortality* | Some concerns | Unknown whether care team was blinded. No protocol for anticoagulant, antiarrhythmic use. |
| Domain 2: Risk of bias due to deviations from the intended interventions (effect of adhering to intervention)  *Surgery duration, cross-clamp time, cardiopulmonary bypass time, reoperation for bleeding* | Low risk | No concerns detected |
| Domain 3: Missing outcome data  *Short-term outcomes: Early post-operative AF, cardiopulmonary bypass time, cross-clamp, reoperations for bleeding, surgery duration, length of ICU stay, length of hospital stay, anticoagulant use, antiarrhythmic use* | Low risk | Few missing data relative to total population/events |
| Domain 3: Missing outcome data  *Long-term outcomes: incident clinical AF at longest follow-up, stroke/systemic thromboembolism, mortality* | High risk | Large number of patients lost to follow-up relative to number of events of AF at follow-up, and stroke/systemic thromboembolism |
| Domain 4: Risk of bias in measurement of the outcome  *All outcomes* | Low risk | Objective outcomes and standardized method of rhythm assessment |
| Domain 5: Risk of bias in selection of the reported result | Low risk | No protocol, but standard outcomes |
| Overall risk of bias  *All Outcomes* | High risk | |

**Lednev 2017:**

| Methods | Randomized control trial in Russia | |
| --- | --- | --- |
| Inclusion Criteria | - Coronary heart disease with requirement for surgery based on recommendations for coronary bypass surgery from the American College of Cardiology and American Heart Association | |
| Exclusion Criteria | - Previous history of AF - Valvular lesions requiring surgical correction - Atherosclerotic lesions of ≥ 2 vessels requiring simultaneous surgical intervention | |
| Interventions | Group 1: No prophylaxis  versus  Group 2: Amiodarone 5 days before surgery and postoperatively (amiodarone prescribed at 10 mg/kg/day until 6 g in total was prescribed, taken under control of heart rate, Q-Tc interval)  versus  Group 3: Radiofrequency ablation of the pulmonary veins | |
| Population Characteristics | None reported | |
| Outcomes Reported in Manuscript and Definitions | - Early post-operative AF - Incident clinical AF at longest follow-up (atrial fibrillation detected over follow-up period of one year) - Length of hospital stay - Surgery duration - Cardiopulmonary bypass time - Reoperations for bleeding (resternotomy for bleeding) - Mortality (6 months) | |
| Protocol for Antiarrhythmic Use | All patients in group 2 received amiodarone 5 days before surgery and post-operatively. Patients in group 1 and 3 did not receive prophylactic amiodarone. | |
| Protocol for Anticoagulant Use | No protocol | |
| Outcomes Clarified by Contacting Authors | Authors contacted regarding baseline characteristics. No reply received. | |
| Potential Conflicts | Funding source: Not reported  Declarations of interest: The authors declare none | |
| Notes | Full text in Russian | |
| ***Risk of bias*** | | |
| *Bias domain* | *Authors’ judgement* | *Support for judgement* |
| Domain 1: Risk of bias arising from the randomization process | Some concerns | No description of randomization process, however no baseline differences noticed |
| Domain 2: Risk of bias due to deviations from the intended interventions (effect of assignment to intervention) | Low risk | Care team was not blinded, however protocol for antiarrhythmic use |
| Domain 2: Risk of bias due to deviations from the intended interventions (effect of assignment to intervention)  *All outcomes* | Low risk | Care team was not blinded, however protocol for antiarrhythmic use |
| Domain 3: Missing outcome data | Low risk | All patients accounted for and underwent rhythm assessment |
| Domain 4: Risk of bias in measurement of the outcome  *Early post-operative AF, incident clinical AF at longest follow-up* | Some concerns | No method of rhythm assessment reported |
| Domain 4: Risk of bias in measurement of the outcome  *Cardiopulmonary bypass time, reoperation for bleeding, surgery duration, length of hospital stay, mortality* | Low risk | Objective outcomes |
| Domain 5: Risk of bias in selection of the reported result | Low risk | No protocol but standard outcomes reported |
| Overall risk of bias  *All Outcomes* | Some concerns | |

**Revishvili 2020:**

| Methods | Single-centre randomized control trial in Russia | |
| --- | --- | --- |
| Inclusion Criteria | - Signed informed consent form - Ischemic heart disease - Critical lesions of coronary arteries with anatomy amenable to coronary artery bypass grafting | |
| Exclusion Criteria | - Presence of AF - Lesions of heart valves - MI within 30 days of hospitalization - Stage III chronic kidney disease and above - Decompensation diabetes mellitus with glycated hemoglobin 7.5% or more | |
| Interventions | No prophylaxis  versus  Radiofrequency ablation of the pulmonary veins | |
| Population Characteristics | Ablation:   - Age (mean, SD): 60.3 ± 6.6 years - Male (%, n): 89.7% (26/29) - Hypertension (%, n): 100.0% (29/29) - Diabetes (%, n): 34.5% (10/29) - Preoperative beta-blocker (%, n): 93.1% (27/29) - COPD (%, n): 10.3% (3/29) - History of myocardial infarction (%, n): 65.5% (19/29) - History of stroke (%, n): 6.9% (2/29) - History of peripheral vascular disease (%, n): 10.3% (3/29)   Control:   - Age (mean, SD): 61.9 ± 6.6 years - Male (%, n): 82.4% (28/34) - Hypertension (%, n): 97.1% (33/34) - Diabetes (%, n): 38.2% (13/34) - Preoperative beta-blocker (%, n): 97.1% (33/34) - COPD (%, n): 11.4% (3/34) - History of myocardial infarction (%, n): 67.6% (23/34) - History of stroke (%, n): 8.8% (3/34) - History of peripheral vascular disease (%, n): 11.8% (4/34) | |
| Outcomes Reported in Manuscript and Definitions | - Early post-operative AF (post-operative atrial fibrillation; episodes longer than 5 minutes considered significant) - Length of hospital stay - Length of intensive care unit stay - Surgery duration - Cross-clamp time - Cardiopulmonary bypass time - Mortality (in-hospital) | |
| Protocol for Antiarrhythmic Use | Amiodarone was provided to patients in atrial fibrillation according to the “accepted scheme” | |
| Protocol for Anticoagulant Use | No protocol | |
| Outcomes Clarified by Contacting Authors | Did not contact authors | |
| Potential Conflicts | Funding source: Trial had no sponsorship  Declarations of interest: The authors declare none | |
| Notes | Paper in Russian, NCT03857711. This is a paper reporting on the results of a pilot study, while Revishvili 2023^4^ is an abstract of the full study. | |
| ***Risk of bias*** | | |
| *Bias domain* | *Authors’ judgement* | *Support for judgement* |
| Domain 1: Risk of bias arising from the randomization process | Some concerns | No randomization method specified, and no clinical parameters reported |
| Domain 2: Risk of bias due to deviations from the intended interventions (effect of assignment to intervention) | Low risk | Care team was not blinded, however protocol for antiarrhythmic use |
| Domain 2: Risk of bias due to deviations from the intended interventions (effect of assignment to intervention) | Low risk | Care team was not blinded, however protocol for antiarrhythmic use |
| Domain 3: Missing outcome data | Low risk | All patients accounted for and underwent rhythm assessment |
| Domain 4: Risk of bias in measurement of the outcome | Low risk | Objective outcomes and standard rhythm assessment method |
| Domain 5: Risk of bias in selection of the reported result | Low risk | Reported outcomes in accordance with protocol |
| Overall risk of bias  *All Outcomes* | Some concerns | |

**Revishvili 2023:**

| Methods | Multi-centre randomized control trial in Russia | |
| --- | --- | --- |
| Inclusion Criteria | - Signed informed consent form - Indications for two or more coronary arteries to undergo bypass surgery | |
| Exclusion Criteria | - Acute coronary syndrome - Previous CABG surgery - Ejection fraction < 40% - Heart valve disease requiring surgical repair - Atrial fibrillation (long-standing persistent, persistent, or paroxysmal) - Respiratory failure - Left ventricular aneurysm requiring surgical correction - Chronic renal failure (≤ 60 mL/min/1 • 73 m^2^) - Chronic diseases heavily impacting prognosis of life (e.g. cancer) - Participation in other trials | |
| Interventions | Group 1: No prophylaxis  versus  Group 2: Radiofrequency ablation of the pulmonary veins  versus  Group 3: Radiofrequency ablation of the pulmonary veins and amiodarone  versus  Group 4: Amiodarone | |
| Population Characteristics: | Group 1:   - Age (mean, SD): 62 ± 6.4 years - Male (%, n): 82.9% (39/47)   Group 2:   - Age (mean, SD): 59.8 ± 6.4 years - Male (%, n): 90.9% (40/44)   Group 3:   - Age (mean, SD): 61.7 ± 6.1 years - Male (%, n): 78.5% (33/42)   Group 4:   - Age (mean, SD): 61.5 ± 6.3 years - Male (%, n): 76.1% (32/42) | |
| Outcomes Reported in Manuscript and Definitions | - Early post-operative AF - Incident clinical AF at longest follow-up - Mortality (long-term mortality) | |
| Protocol for Antiarrhythmic Use | All patients in group 3 and 4 received amiodarone. According to Revishvili 2020, amiodarone was provided to patients in atrial fibrillation according to the “accepted scheme^5^” | |
| Protocol for Anticoagulant Use | No protocol | |
| Outcomes Clarified by Contacting Authors | Authors contacted regarding incident clinical AF at longest follow-up (atrial fibrillation/atrial flutter at 1 year) | |
| Potential Conflicts | Funding source: Not reported  Declarations of interest: Not reported | |
| Notes | Abstract in Russian, NCT03857711. This is an abstract of the PULVAB trial, while Revishvili 2020^5^ is a paper presenting results of a pilot study. | |
| ***Risk of bias*** | | |
| *Bias domain* | *Authors’ judgement* | *Support for judgement* |
| Domain 1: Risk of bias arising from the randomization process | Some concerns | No randomization method and no clinical parameters reported |
| Domain 2: Risk of bias due to deviations from the intended interventions (effect of assignment to intervention) | Low risk | Care team was not blinded, however protocol for antiarrhythmic use according to Revishvili 2020^5^ |
| Domain 2: Risk of bias due to deviations from the intended interventions (effect of assignment to intervention) | Low risk | Care team was not blinded, however protocol for antiarrhythmic use according to Revishvili 2020^5^ |
| Domain 3: Missing outcome data  *Short-term outcomes: Early post-operative AF* | Low risk | All patients accounted for and underwent rhythm assessment |
| Domain 3: Missing outcome data  *Long-term outcomes: incident clinical AF at longest follow-up, mortality* | High risk | Substantial number of patients missing at one year follow-up (26/175; 14.9%) |
| Domain 4: Risk of bias in measurement of the outcome  *Early post-operative AF, incident clinical AF at longest follow-up* | Low risk | Standard rhythm assessment method |
| Domain 5: Risk of bias in selection of the reported result | High risk | Several outcomes in protocol not reported on (stroke and/or systemic thromboembolism, cardiopulmonary bypass time, cross-clamp time, length of hospital stay, length of intensive care unit stay) |
| Overall risk of bias  *All Outcomes* | High risk | |

**Teijeira 2014:**

| Methods | Single-centre randomized control trial in Quebec, Canada | |
| --- | --- | --- |
| Inclusion Criteria | - Age > 65 years old - Undergoing CABG surgery - History of hypertension or diabetes | |
| Exclusion Criteria | - Prior thoracic surgery - History of atrial fibrillation | |
| Interventions | No prophylaxis  versus  Radiofrequency ablation of the pulmonary veins | |
| Population Characteristics | Ablation:   - Age (mean, SD): 71.6 ± 4.6 years - Male (%, n): 88.0% (22/25) - Hypertension (%, n): 92.0% (23/25) - Diabetes (%, n): 40.0% (10/25) - Preoperative beta-blocker (%, n): 96.0% (24/25)   Control:   - Age (mean, SD): 71.6 ± 4.6 years - Male (%, n): 80.0% (20/25) - Hypertension (%, n): 92.0% (23/25) - Diabetes (%, n): 48.0% (12/25) - Preoperative beta-blocker (%, n): 100% (25/25) | |
| Outcomes Reported in Manuscript and Definitions | - Early post-operative AF (post-operative atrial fibrillation lasting two minutes or longer) - Incident clinical AF at longest follow-up (atrial fibrillation or atrial flutter at 24 months) - Antiarrhythmic use (amiodarone) - Anticoagulant use (warfarin) - Stroke (during immediate postoperative period) - Length of hospital stay - Length of intensive care unit stay - Surgery duration - Cross clamp time - Cardiopulmonary bypass time - Reoperations for bleeding - Mortality (24 months) - Left atrial flutter | |
| Protocol for Antiarrhythmic Use | No protocol | |
| Protocol for Anticoagulant Use | No protocol | |
| Outcomes Clarified by Contacting Authors | Length of hospital stay, length of ICU stay, cross-clamp time, and cardiopulmonary bypass time | |
| Potential Conflicts | Funding source: Trial had no sponsorship  Declarations of interest: The authors declare none | |
| Notes | Paper in Spanish | |
| ***Risk of bias*** | | |
| *Bias domain* | *Authors’ judgement* | *Support for judgement* |
| Domain 1: Risk of bias arising from the randomization process | Some concerns | No randomization method specified, however baseline parameters are balanced |
| Domain 2: Risk of bias due to deviations from the intended interventions (effect of assignment to intervention)  *Antiarrhythmic use, anticoagulant use* | High risk | Caregivers were not blinded and no protocol for antiarrhythmic or anticoagulant use. |
| Domain 2: Risk of bias due to deviations from the intended interventions (effect of assignment to intervention)  *Early post-operative AF, incident clinical AF at longest follow-up, stroke, length of hospital stay, length of intensive care unit stay mortality* | Some concerns | Caregivers were not blinded and no protocol for antiarrhythmic or anticoagulant use. |
| Domain 2: Risk of bias due to deviations from the intended interventions (effect of assignment to intervention)  *Surgery duration, cross-clamp time, cardiopulmonary bypass time, reoperations for bleeding* | Low risk | No concerns detected |
| Domain 2: Risk of bias due to deviations from the intended interventions (effect of adhering to intervention)  *Antiarrhythmic use, anticoagulant use* | High risk | Caregivers were not blinded and no protocol for antiarrhythmic or anticoagulant use. |
| Domain 2: Risk of bias due to deviations from the intended interventions (effect of adhering to intervention)  *Early post-operative AF, incident clinical AF at longest follow-up, stroke, length of hospital stay, length of intensive care unit stay, mortality* | Some concerns | Caregivers were not blinded and no protocol for antiarrhythmic or anticoagulant use. |
| Domain 2: Risk of bias due to deviations from the intended interventions (effect of adhering to intervention)  *Surgery duration, cross-clamp time, cardiopulmonary bypass time, reoperations for bleeding, anticoagulant use* | Low risk | No concerns detected |
| Domain 3: Missing outcome data  *Long-term outcomes: incident clinical AF at longest follow-up, stroke, mortality* | High risk | Substantial number of missing patients at 24-month follow up (8/50 (16%) patients lost to follow-up) |
| Domain 3: Missing outcome data  *Short-term outcomes: Early post-operative AF, antiarrhythmic use, anticoagulant use, length of hospital stay, length of intensive care unit stay, surgery duration, cross clamp time, cardiopulmonary bypass time, reoperations for bleeding* | Low risk | All patients accounted for |
| Domain 4: Risk of bias in measurement of the outcome | Low risk | Objective outcomes and standard method of rhythm detection |
| Domain 5: Risk of bias in selection of the reported result | Low risk | No protocol but standard outcomes reported |
| Overall risk of bias  *Incident clinical AF at longest follow-up, antiarrhythmic use, anticoagulant use, stroke, mortality* | High risk | |
| Overall risk of bias  *Early post-operative AF, Surgery duration, cross-clamp time, cardiopulmonary bypass time, reoperations for bleeding, length of hospital stay, length of intensive care unit stay* | Some concerns | |

**Willekes 2023:**

| Methods | Single-centre randomized control trial in Michigan, United States | |
| --- | --- | --- |
| Inclusion Criteria | - > 70 years old - Undergoing CABG, aortic valve replacement or combined CABG/aortic valve replacement | |
| Exclusion Criteria | - Emergency surgery - Reoperation - Mitral valve disease or mitral procedure - Ejection fraction < 35% - Left atrial enlargement of any degree - Permanent pacemaker - History of antiarrhythmics Class I or III - Atrial or ventricular dysrhythmia history | |
| Interventions | No prophylaxis  versus  Radiofrequency ablation of the pulmonary veins | |
| Population Characteristics | Ablation:   - Age (mean, SD): 75 ± 4 years - Male (%, n): 77.4% (24/31) - CHA2DS2-VASc score (mean, SD): 4 ± 1 - LA size (median, IQR): 27.9 [23.0-30.0] ml/m^2^ - BMI (mean, SD): 29.3 ± 4.2 - Hypertension (%, n): 87.1% (27/31) - Diabetes (%, n): 48.4% (15/31) - Preoperative beta-blocker (%, n): 100% (31/31) - Preoperative hemoglobin: 13.8 ± 1.2 g/dl - COPD (%, n): 9.7% (3/31) - History of peripheral vascular disease (%, n): 29.0% (9/31)   Control:   - Age (mean, SD): 75 ± 4 years - Male (%, n): 51.7% (15/29) - CHA2DS2-VASc score (mean, SD): 4 ± 1 - LA size (median, IQR): 24.5 [20.0-27.5] ml/m^2^ - BMI (mean, SD): 29.2 ± 4.9 - Hypertension (%, n): 89.7% (26/29) - Diabetes (%, n): 27.6% (8/29) - Preoperative beta-blocker (%, n): 100% (29/29) - Preoperative hemoglobin: 13.6 ± 1.2 g/dl - COPD (%, n): 13.8% (4/29) - History of peripheral vascular disease (%, n): 27.6% (8/29) | |
| Outcomes Reported in Manuscript and Definitions | - Early post-operative AF (irregular heart rhythm without P waves > 30 seconds; post-operative atrial flutter) - Incident clinical AF at longest follow-up (atrial fibrillation or flutter at 12 months) - Antiarrhythmic use (primarily amiodarone with or without diltiazem) - Anticoagulant use - Stroke - Length of hospital stay - Surgery duration - Cross clamp time - Cardiopulmonary bypass time - Reoperations for bleeding - Mortality (1 year) - Permanent pacemaker implantation | |
| Protocol for Antiarrhythmic Use | Treated with antiarrhythmic agent (primarily amiodarone with or without diltiazem) therapy after 24 hours of atrial fibrillation or 2 or more episodes of atrial fibrillation | |
| Protocol for Anticoagulant Use | Treated with anticoagulant therapy after 24 hours of atrial fibrillation or 2 or more episodes of atrial fibrillation | |
| Outcomes Clarified by Contacting Authors | Did not contact authors | |
| Potential Conflicts | Funding source: Spectrum Health Department of Cardiothoracic Service Line  Declarations of interest: The authors declare none | |
| Notes | NCT03604432 | |
| ***Risk of bias*** | | |
| *Bias domain* | *Authors’ judgement* | *Support for judgement* |
| Domain 1: Risk of bias arising from the randomization process | Low risk | Used computer generated sequence |
| Domain 2: Risk of bias due to deviations from the intended interventions (effect of assignment to intervention) | High risk | 2/31 (6.5%) patients in intervention group were excluded from analysis post-randomization |
| Domain 2: Risk of bias due to deviations from the intended interventions (effect of adhering to intervention) | Low risk | Care team was not blinded, however protocol for antiarrhythmic and anticoagulant use |
| Domain 3: Missing outcome data  *Incident clinical AF at longest follow-up, stroke, antiarrhythmic use, anticoagulant use, early post-operative AF, mortality* | High risk | 2/31 (6.5%) patients were excluded from intervention group, and there were few events |
| Domain 3: Missing outcome data  *Length of hospital stay, cross clamp time, cardiopulmonary bypass time* | Low risk | Small proportion of missing data |
| Domain 4: Risk of bias in measurement of the outcome  *Length of hospital stay, cross clamp time, cardiopulmonary bypass time, stroke, antiarrhythmic use, anticoagulant use, early post-operative AF* | Low risk | Objective outcomes and consistent rhythm monitoring protocol |
| Domain 4: Risk of bias in measurement of the outcome  *Incident clinical AF at longest follow-up* | Some concerns | No protocol for identification of AF at follow-up and participants were not blinded |
| Domain 5: Risk of bias in selection of the reported result | Low risk | No concerns detected |
| Overall risk of bias  *All outcomes* | High risk | |

**Appendix 5: Outcomes Reported in Included Trials**

| **Study** | **Outcomes Reported** |
| --- | --- |
| Al-Atassi 2014 | - Early post-operative AF - Antiarrhythmic use - Anticoagulant use - Stroke - Length of hospital stay |
| Kiaii 2015 | - Early post-operative AF - Incident clinical AF at longest follow-up - Antiarrhythmic use - Anticoagulant use - Stroke - Length of hospital stay - Length of intensive care unit stay - Surgery duration - Cross-clamp time - Cardiopulmonary bypass time - Reoperations for bleeding - Mortality |
| Lednev 2017 | - Early post-operative AF - Incident clinical AF at longest follow-up - Length of hospital stay - Surgery duration - Cardiopulmonary bypass time - Reoperations for bleeding - Mortality |
| Revishvili 2020 | - Early post-operative AF - Length of hospital stay - Length of intensive care unit stay - Surgery duration - Cross-clamp time - Cardiopulmonary bypass time - Mortality |
| Revishvili 2023 | - Early post-operative AF - Incident clinical AF at long-term follow up - Mortality |
| Teijeira 2014 | - Early post-operative AF - Incident clinical AF at longest follow-up - Antiarrhythmic use - Anticoagulant use - Stroke - Length of hospital stay - Length of intensive care unit stay - Surgery duration - Cross clamp time - Cardiopulmonary bypass time - Reoperations for bleeding - Mortality - Left atrial flutter |
| Willekes 2023 | - Early post-operative AF - Incident clinical AF at longest follow-up - Antiarrhythmic use - Anticoagulant use - Stroke - Length of hospital stay - Surgery duration - Cross clamp time - Cardiopulmonary bypass time - Reoperations for bleeding - Mortality - Permanent pacemaker implantation |

**Appendix 6: Sources of Risk of Bias in Included Trials:**

| Domain | Sources of Risk of Bias |
| --- | --- |
| Domain 1: Risk of bias arising from the randomization process | - We judged five of the seven trials as having “some concerns” for risk of bias arising from the randomization process. These trials did not describe their randomization procedures in detail, although there were no obvious between-group imbalances. |
| Domain 2: Risk of bias due to deviations from the intended interventions | - We judged three trials as “high risk” for antiarrhythmic use and three trials as “high risk” for anticoagulant use, due to not being blinded, and lacking protocols for antiarrhythmic and anticoagulant use, respectively. - We judged the risk of bias for all other related outcomes as at least “some concerns” for such trials. - We judged two trials to be at “high risk” for deviations from the intended interventions for all outcomes due to large number of post-randomization exclusion. |
| Domain 3: Missing outcome data | - We rated three trials as “high risk” of bias due to missing data for long-term outcomes (incident clinical AF at longest follow-up and stroke) resulting from substantial loss to follow-up - We rated one trial as “high risk” of bias for all dichotomous outcomes (early post-operative AF, long-term AF, antiarrhythmic use, and anticoagulant use) due to substantial exclusion post-randomization and few events |
| Domain 4: Risk of bias in measurement of the outcome | - We judged one trial as having “some concerns” for bias arising due to measurement of early post-operative AF and incident clinical AF at longest follow-up, as it did not report the method of rhythm assessment. |
| Domain 5: Risk of bias in selection of the reported result | - We judged one trial as being at “high risk” for reporting bias, since the published abstract did not report on several outcomes described in the registered protocol. |

**Appendix 7: Subgroup Analyses**

**Risk of Bias, Early Post-operative AF:**

###
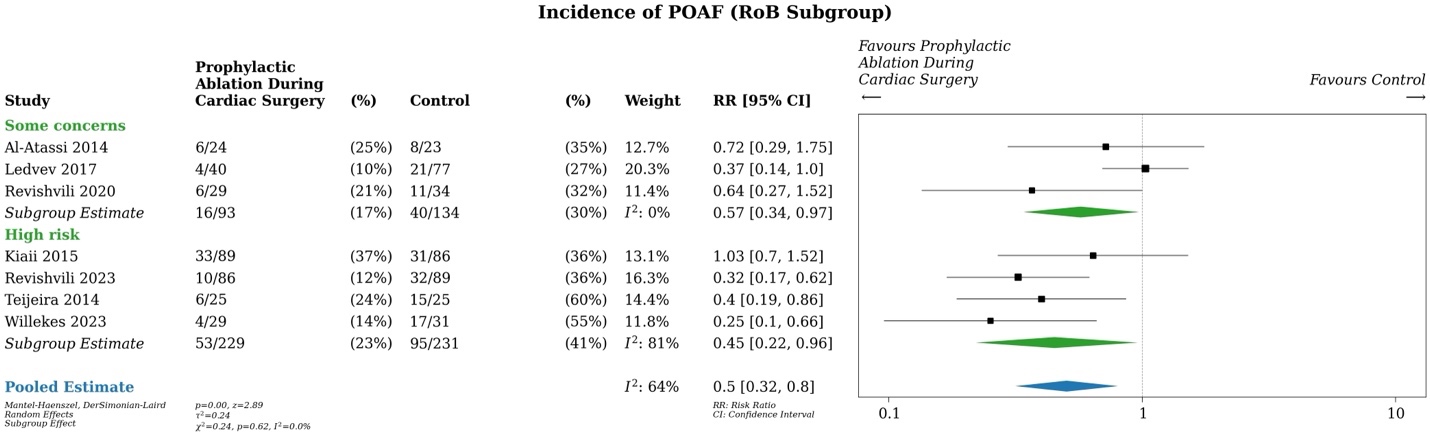


**Risk of Bias, Incident Clinical AF at Longest Follow-up:**

###
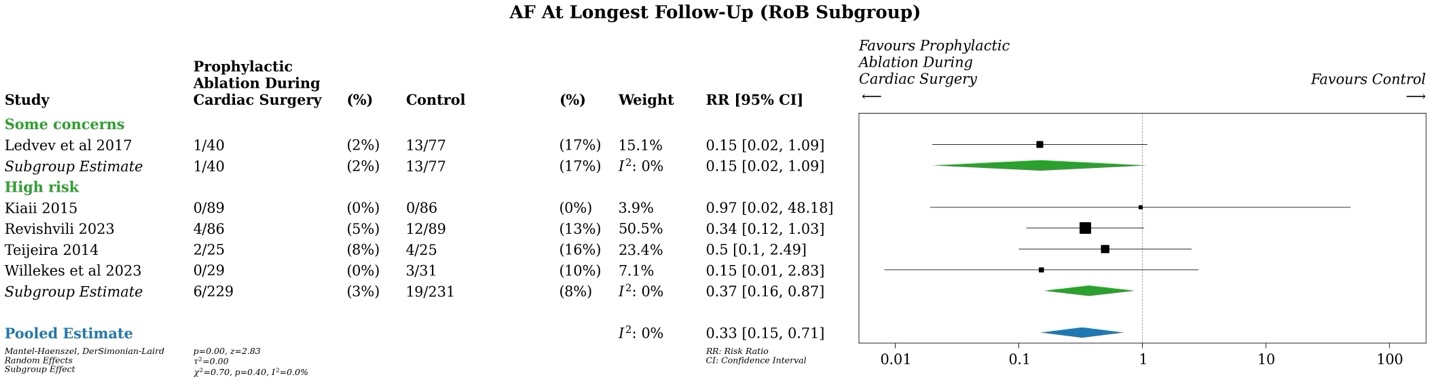


**Ablation Approaches, Early Post-operative AF:**

###
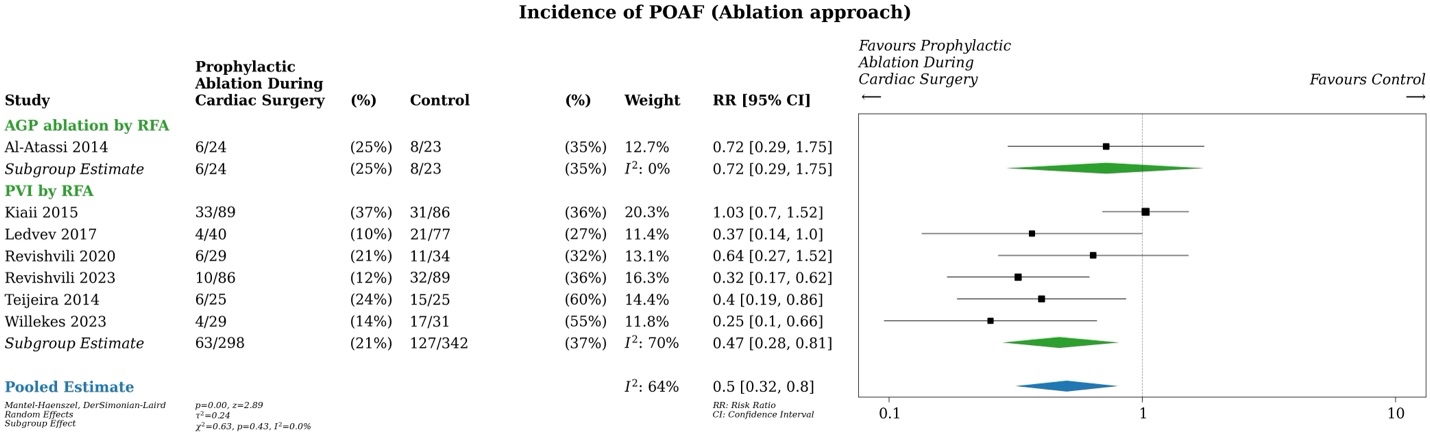


**Method of Rhythm Assessment, Early Post-operative AF:**


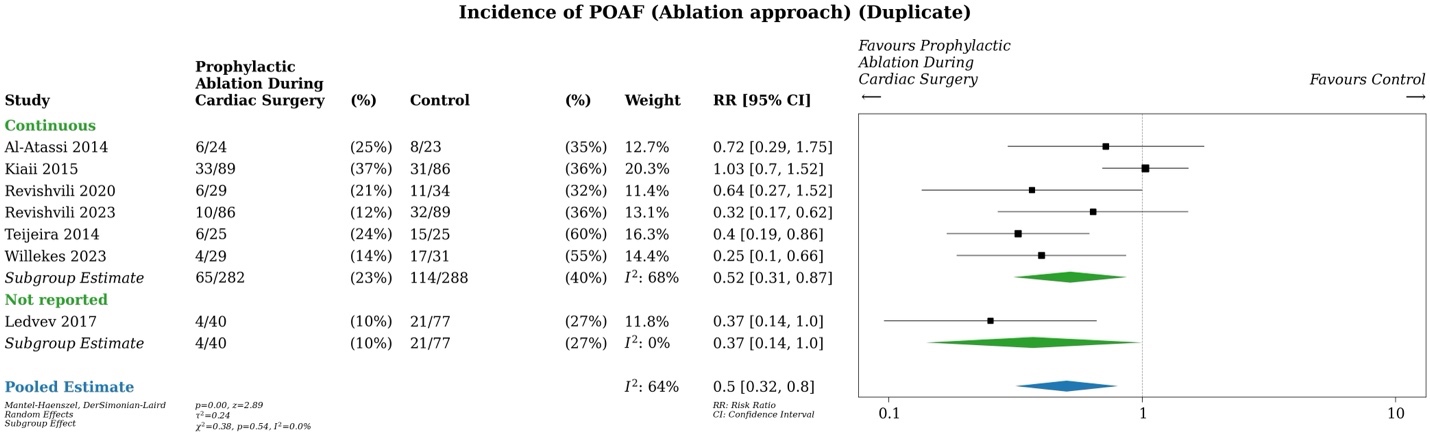


**Method of Rhythm Assessment, Incident Clinical AF at Longest Follow-up:**


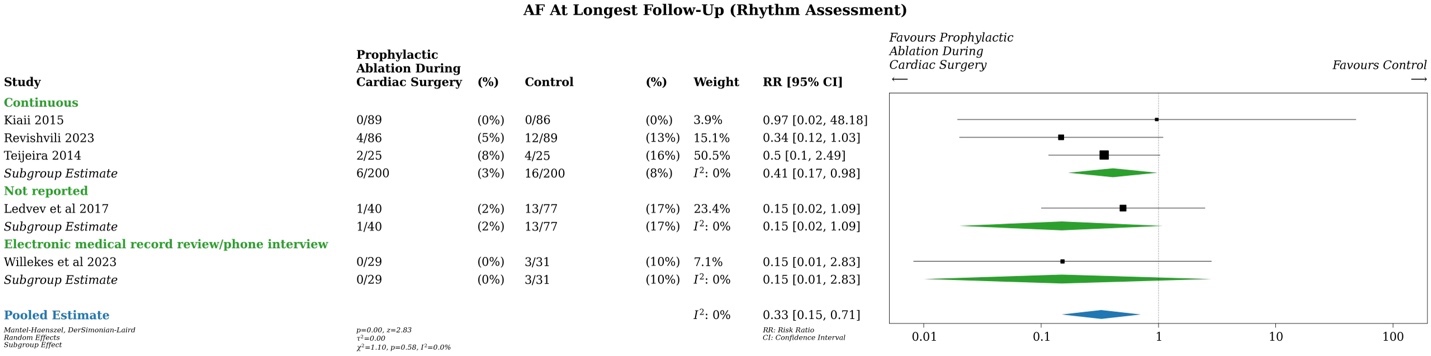


**Amiodarone Used in Comparator Group, Early Post-operative AF:**


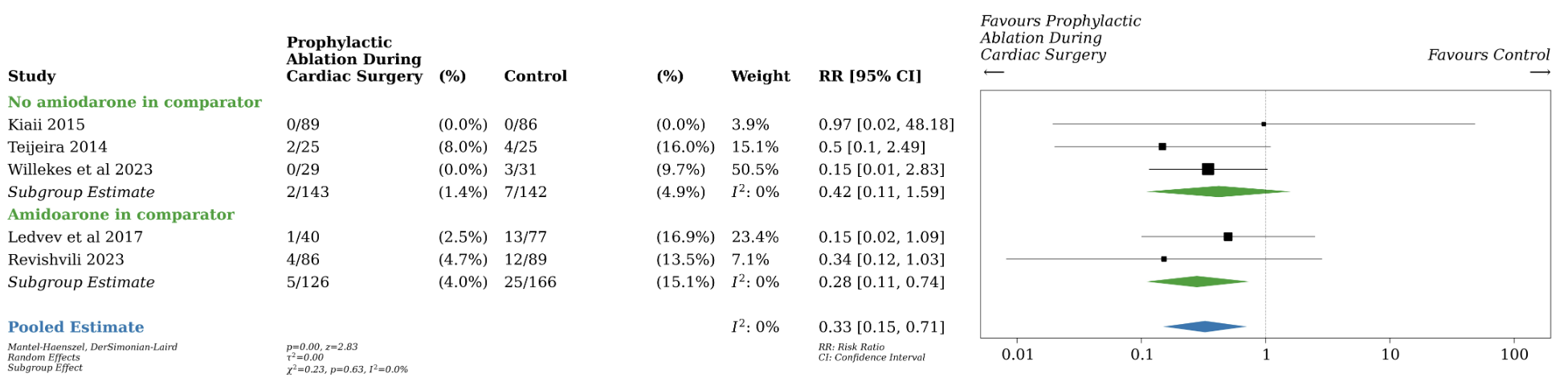


**Amiodarone Used in Comparator Group, Incident Clinical AF at Longest Follow-up:**

##
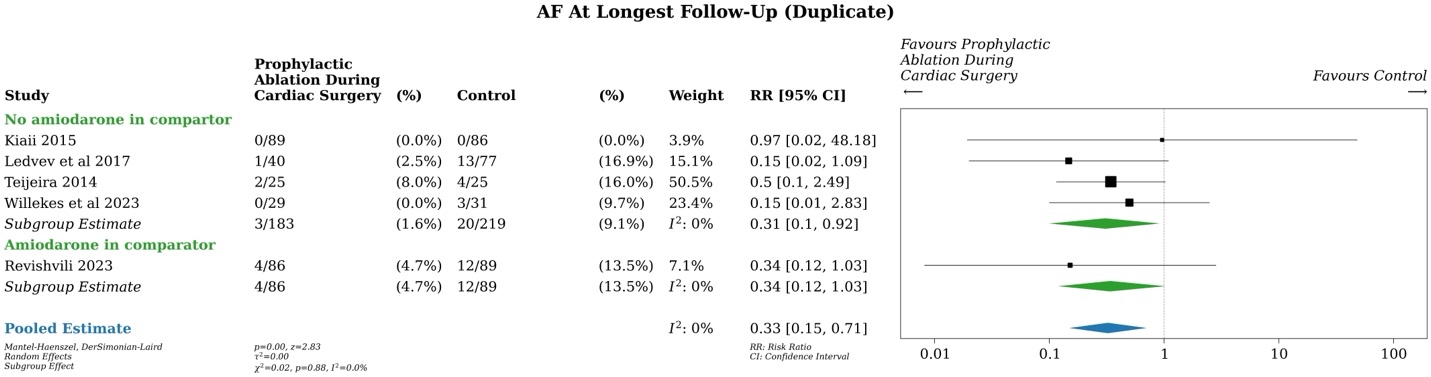


**Amiodarone Used in Comparator Group, Length of Hospital Stay:**


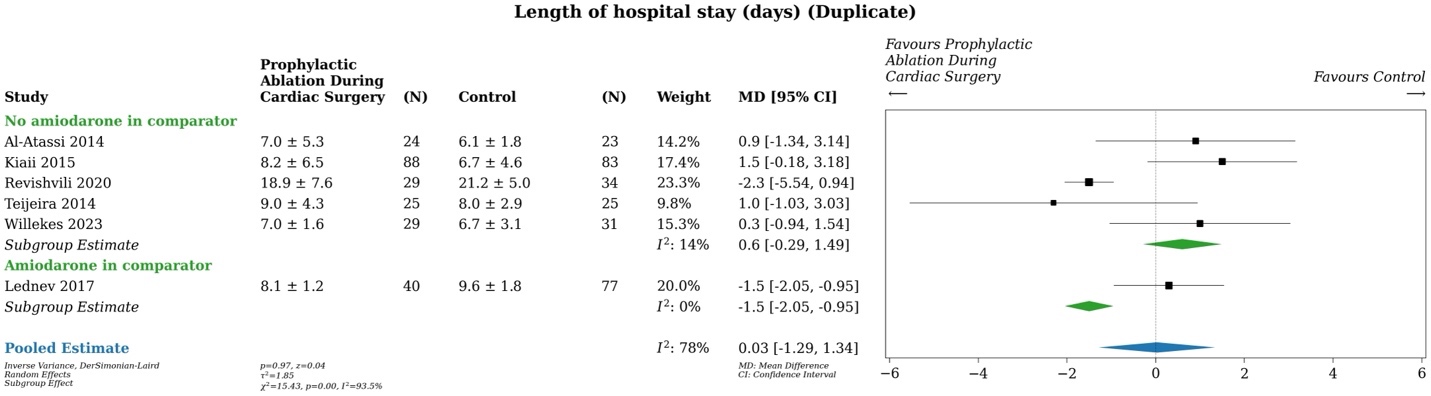


**Protocol for Antiarrhythmic Use, Early Post-operative AF:**


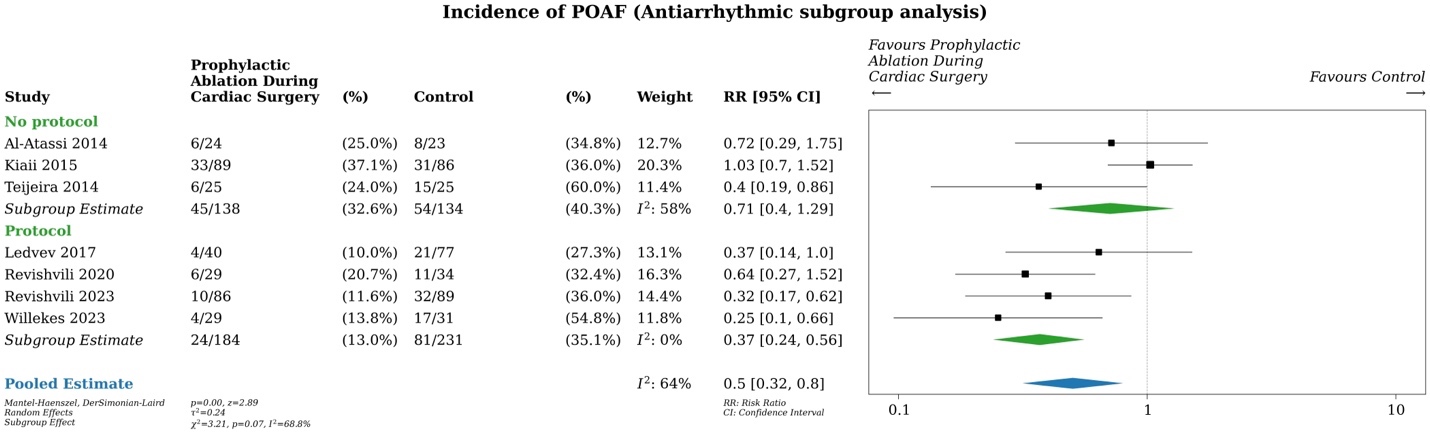


**Protocol for Antiarrhythmic Use, Incident Clinical AF at Longest Follow-up:**


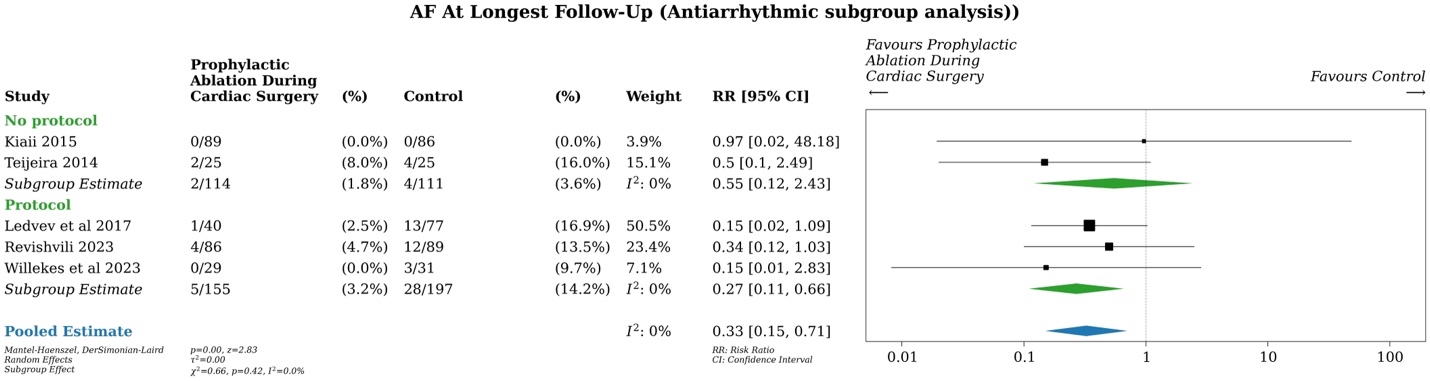


**Protocol for Antiarrhythmic Use, Antiarrhythmic Use:**


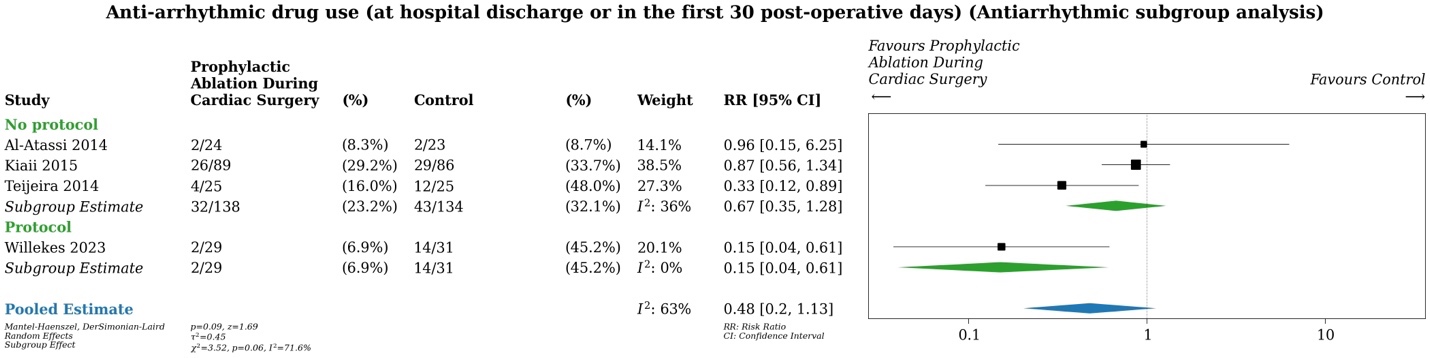


**Protocol for Antiarrhythmic Use, Length of Hospital Stay:**


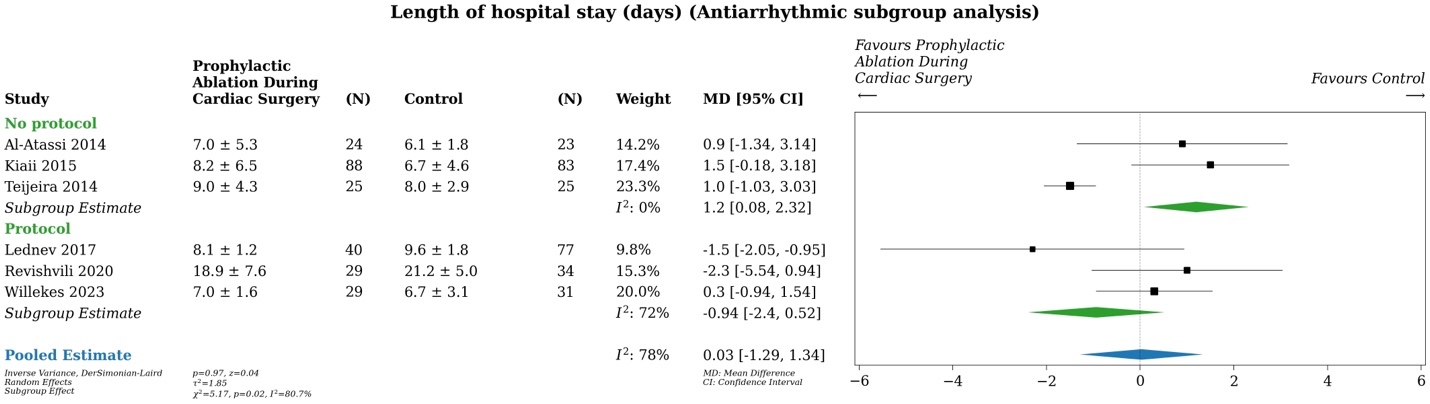


**Protocol for Antiarrhythmic Use, Length of Intensive Care Unit Stay:**
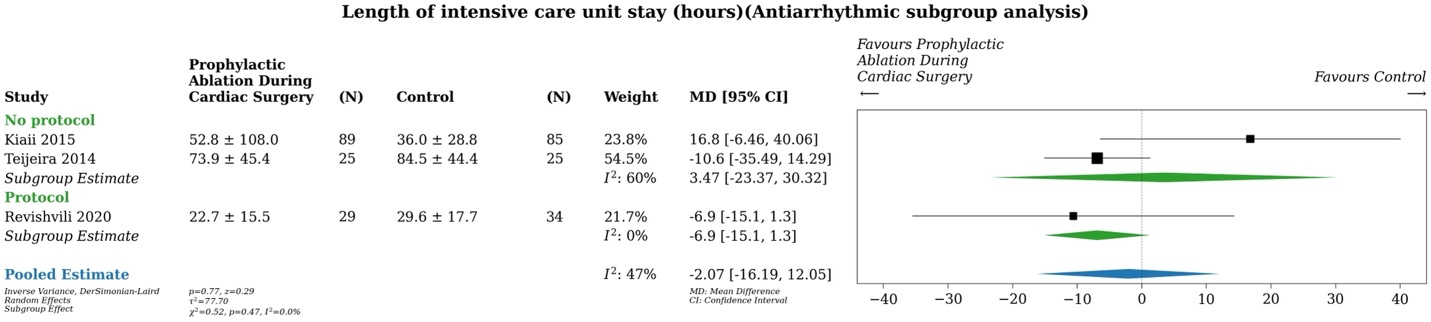


**Protocol for Anticoagulant Use, Early Post-operative AF:**


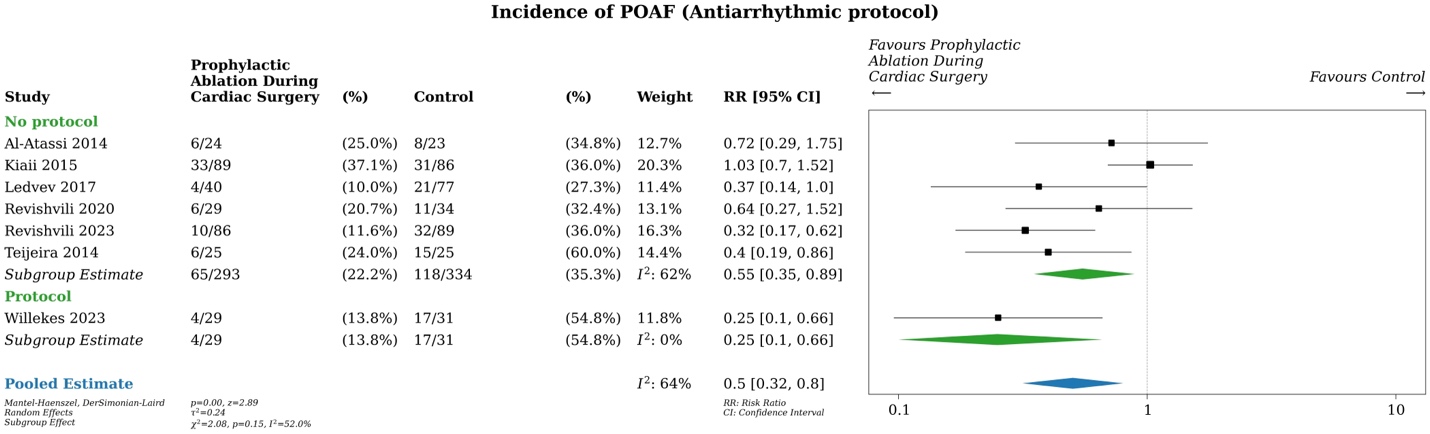


**Protocol for Anticoagulant Use, Incident Clinical AF at Long-term Follow-up:**


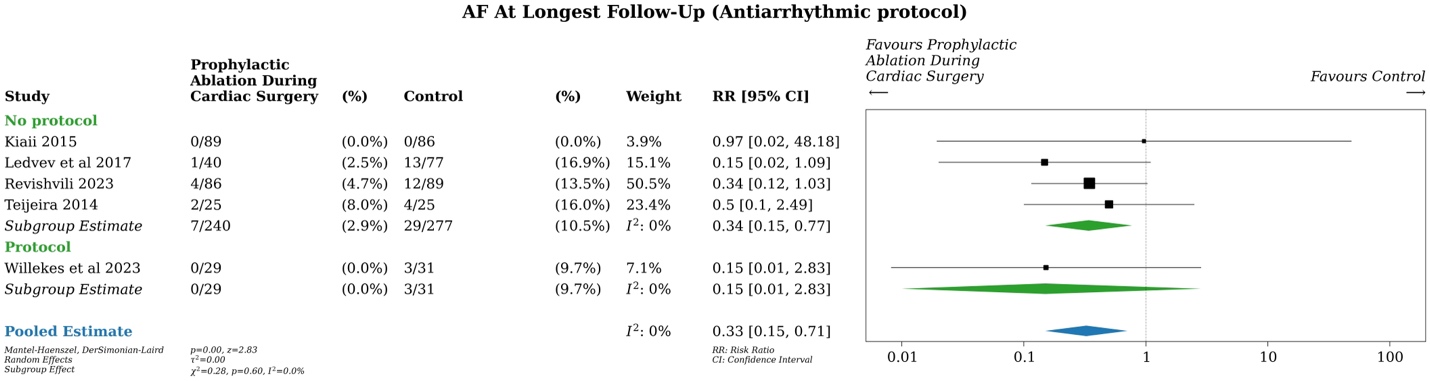


**Protocol for Anticoagulant Use, Length of Hospital stay:**


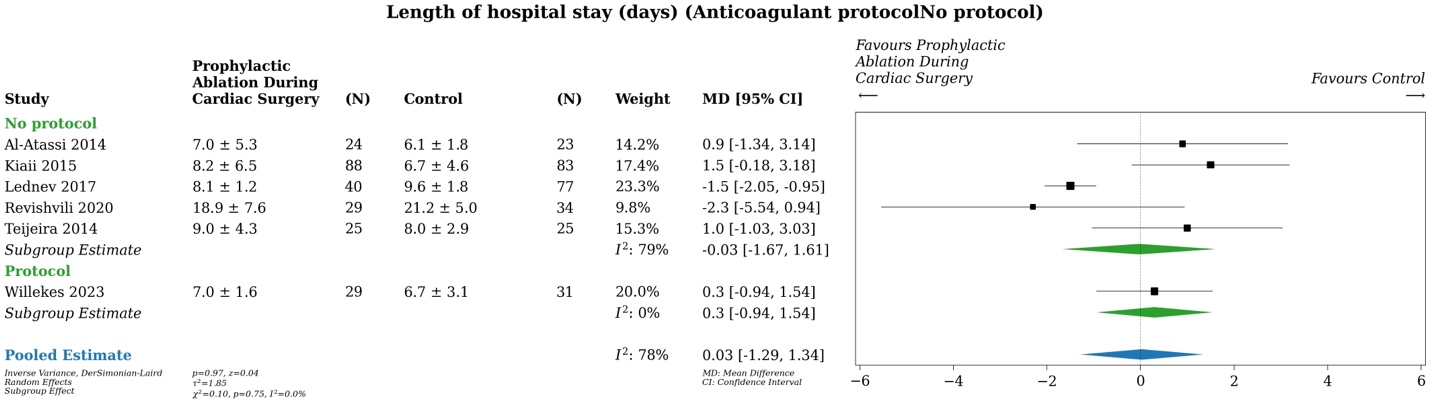


**Protocol for Anticoagulant Use, Anticoagulant Use:**


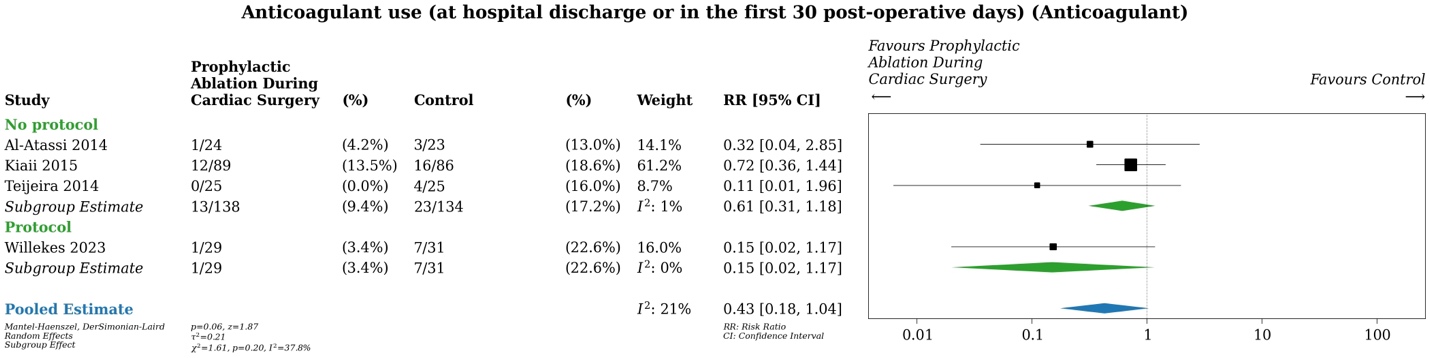


**Protocol for Anticoagulant Use, Stroke and/or Systemic Thromboembolism:**


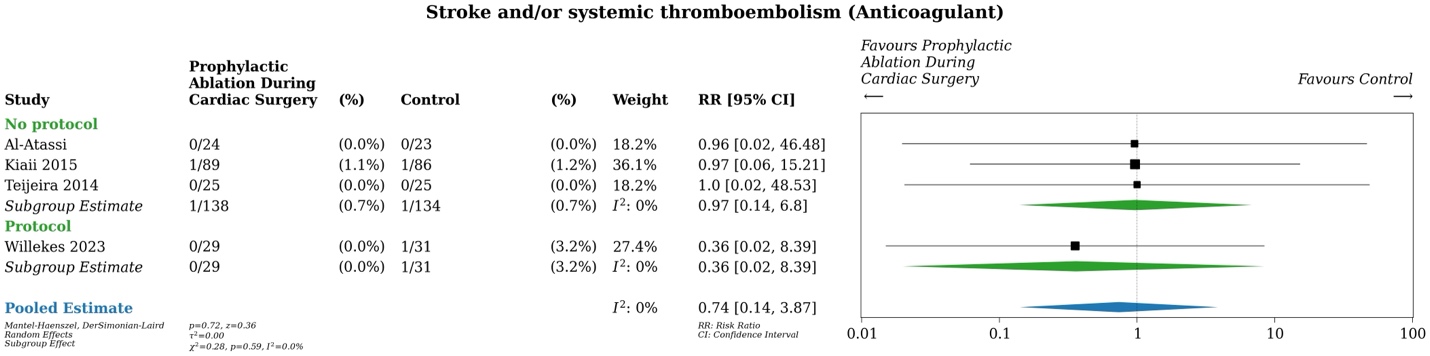

Supplement: ivae195_Supplementary_Data [file ivae195_supplementary_data.zip › VISANJI Supplementary Appendices 1.docx]
