# Supplementary material for: Prophylactic ablation during cardiac surgery in patients without atrial fibrillation: a systematic review and meta-analysis of randomized trials
Source: Interdiscip Cardiovasc Thorac Surg. 2024 Nov 26;39(6):ivae195. doi: 10.1093/icvts/ivae195 (PMC11661978; doi:10.1093/icvts/ivae195)
Supplement: ivae195_Supplementary_Data [file ivae195_supplementary_data.zip › VISANJI Supplementary Appendices 2 .docx]

**Appendix 8. Leave-one-out Meta-analysis**

**Appendix 9. Summary of Meta-Analysis Outcomes**

**Appendix 10. Funnel Plots**

**Appendix 11. Description of Incident Clinical Atrial Fibrillation at Longest Follow-up**

**Appendix 12. Forest plots for Other Outcomes**

**Appendix 13. Description of Antiarrhythmic Use**

**Appendix 8: Leave-one-out Meta-analysis:**

**Early Post-operative AF (Without Al-Atassi 2014):**


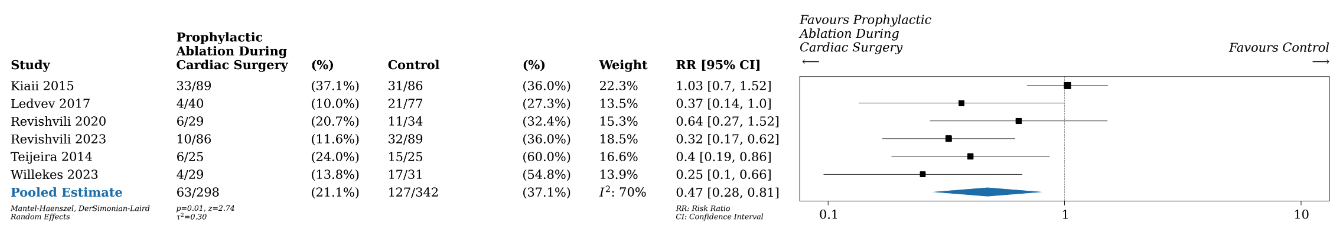


**Early Post-operative AF (Without Kiaii 2015):**


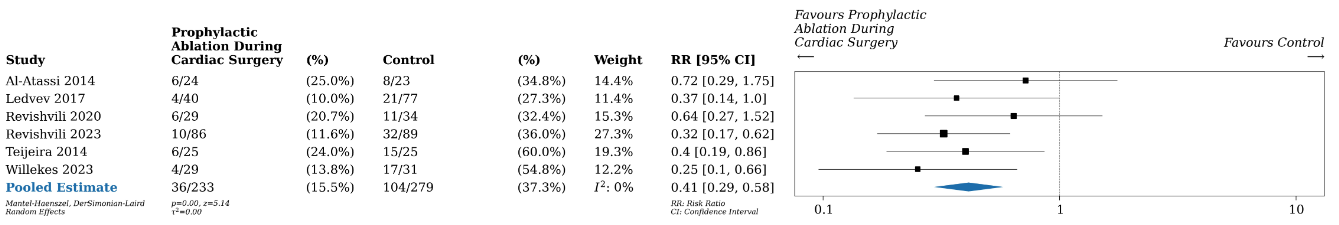


**Early Post-operative AF (Without Ledvev 2017):**


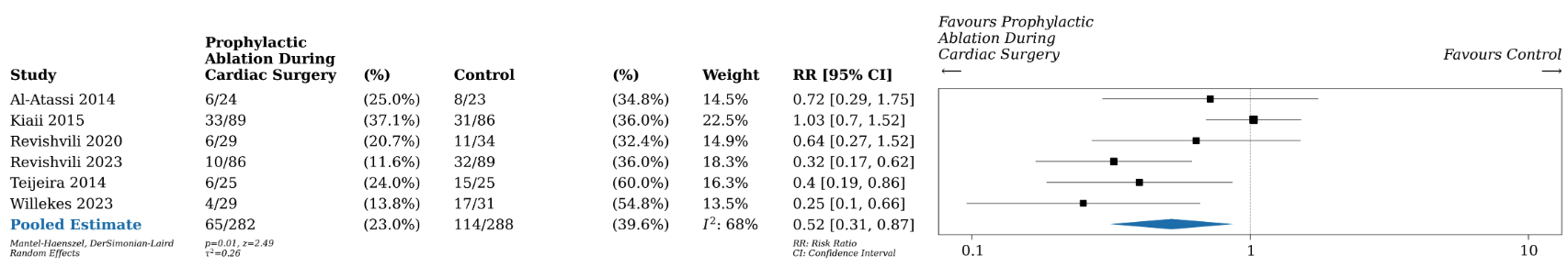


**Early Post-operative AF (Without Revishvili 2020):**

**
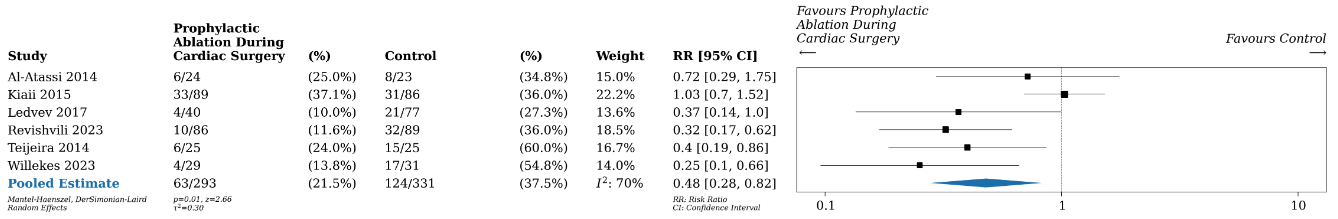
**

**Early Post-operative AF (Without Revishvili 2023):**

**
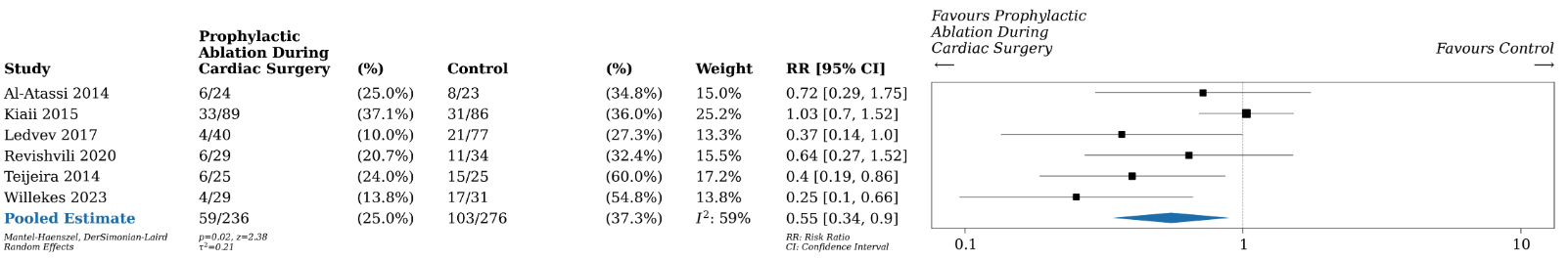
**

**Early Post-operative AF (Without Teijeira 2014):**

**
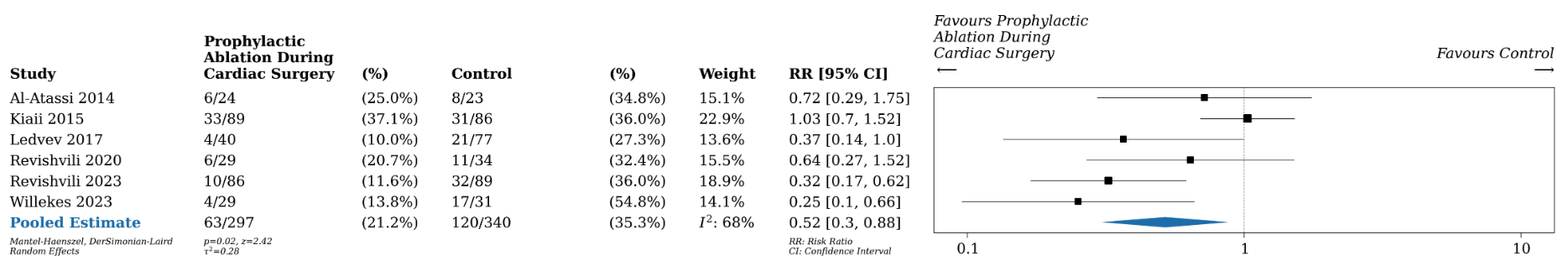
**

**Early Post-operative AF (Without Willekes et al. 2023):**

**
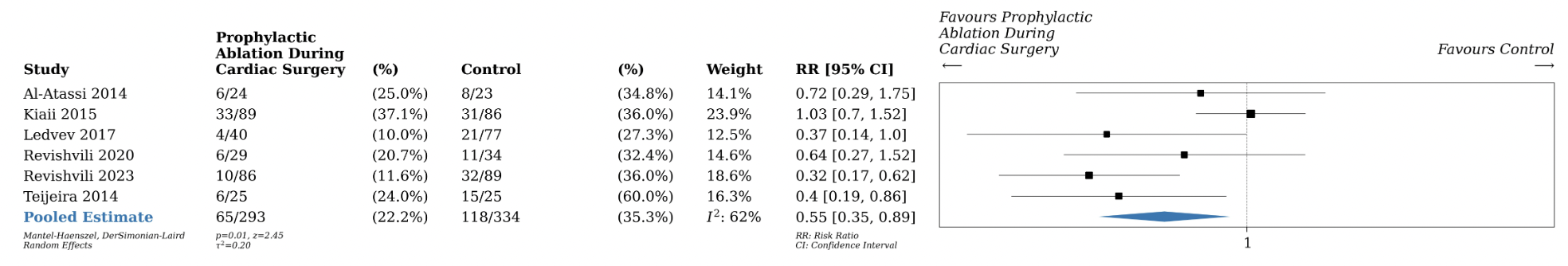
**

**Incident Clinical AF at Longest Follow-up (Without Kiaii et al. 2015):**

**
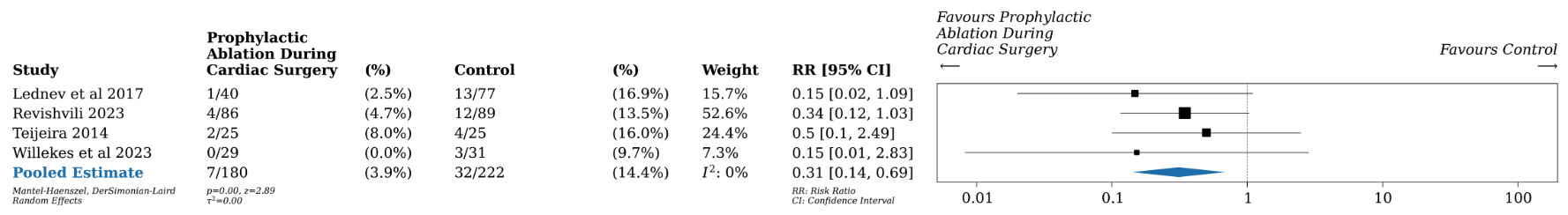
**

**Incident Clinical AF at Longest Follow-up (Without Levnev et al. 2017):**

**
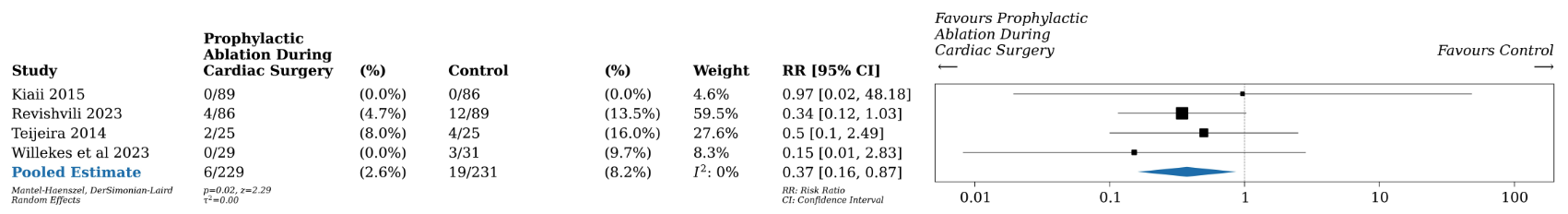
**

**Incident Clinical AF at Longest Follow-up (Without Teijeira et al. 2014):**

**
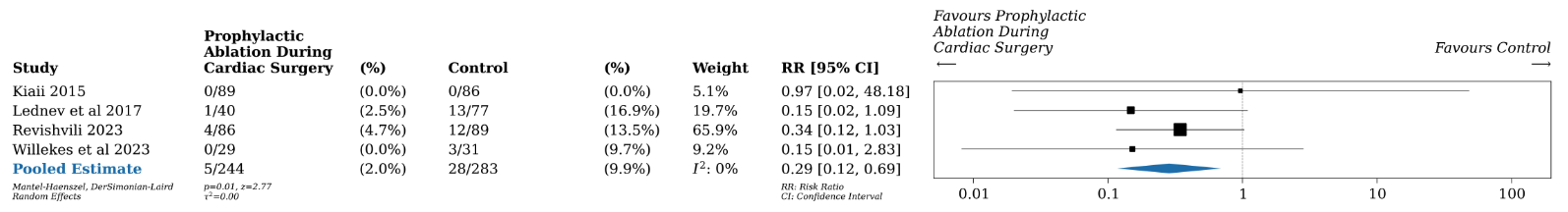
**

**Incident Clinical AF at Longest Follow-up (Without Teijeira et al. 2014):**


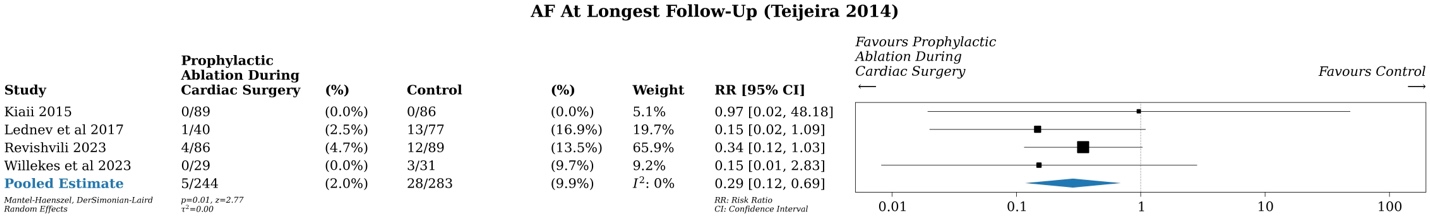


**Incident Clinical AF at Longest Follow-up (Without Willekes et al. 2023):**

**
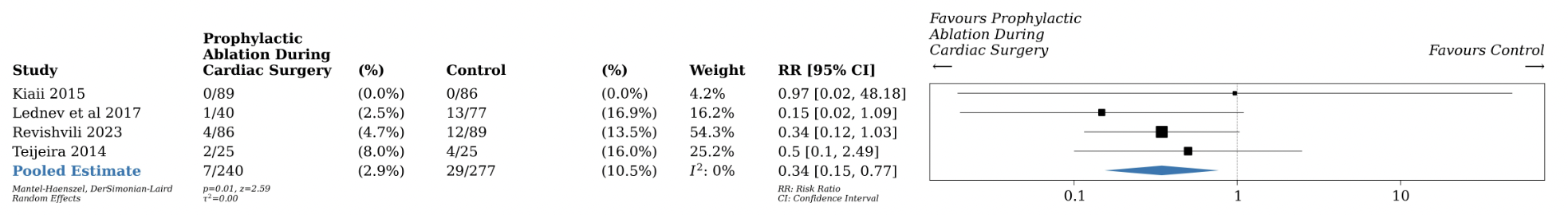
**

**Appendix 9: Summary of Meta-Analysis Outcomes:**

| **Certainty assessment** | | | | | | | | | **№ of patients and**  **events (if applicable)** | | | **Effect size** | | |
| --- | --- | --- | --- | --- | --- | --- | --- | --- | --- | --- | --- | --- | --- | --- |
| **№ of Trials** | **Trial design** | **Risk of bias** | **Inconsistency** | | **Indirectness** | **Imprecision** | **Other considerations** | **Certainty of evidence** | **Ablation** | **Control** | | **Relative risk (95% CI) and ARR**^[[1]](#footnote-1)^ **/mean difference** | ***P* Value** | **I^2^ %** |
| Early Post-operative AF | | | | | | | | | | | | | | |
| 7 | Randomized trials | Serious^a^ | | Serious^b^ | Not serious | Not serious | None | **◯◯**⨁⨁ Low | 69/322  (21.4%) | 135/365 (37.0%) | | **RR 0.50** (0.32 to 0.80),  15.6% ARR | 0.00 | 64 |
| ^a^ all were open label, and three trials lacked structured protocols for antiarrhythmic medications  ^b^ heterogeneity statistics is > 50% and not all confidence intervals overlap | | | | | | | | | | | | | | |
| Incident Clinical AF at Longest Follow-up | | | | | | | | | | | | | | |
| 5 | Randomized trials | Serious^c^ | | Not serious | Serious^d^ | Not serious | None | **◯◯**⨁⨁ Low | 7/269  (2.6%) | 32/308  (10.4%) | | **RR 0.33** (0.15 to 0.71), 7.8% ARR | 0.00 | 0 |
| ^c^ all were open label, and two trials lacked structured protocols for antiarrhythmic medications  ^d^ trials had different definitions/different ascertainment techniques | | | | | | | | | | | | | | |
| Antiarrhythmic use | | | | | | | | | | | | | | |
| 4 | Randomized trials | Serious^a^ | | Serious^e^ | Not serious | Serious^f^ | None | **◯◯◯**⨁ Very low | 34/167  (20.4%) | 57/165  (34.5%) | | **RR 0.48** (0.20 to 1.13), 14.1% ARR | 0.09 | 63 |
| ^a^ all were open label, and three trials lacked structured protocols for antiarrhythmic medications  ^e^ heterogeneity statistics > is 50%  ^f^ significant benefit or harm cannot be excluded | | | | | | | | | | | | | | |
| Anticoagulant use | | | | | | | | | | | | | | |
| 4 | Randomized trials | Serious^g^ | | Not serious | Not serious | Serious^f^ | None | **◯◯**⨁⨁ Low | 14/167  (8.4%) | | 30/165  (18.2%) | **RR 0.43** (0.18 to 1.04), 9.8% ARR | 0.06 | 21 |
| ^g^ all were open label, and three trials lacked structures protocols for anticoagulant medications  ^f^ significant benefit or harm cannot be excluded | | | | | | | | | | | | | | |
| Length of Hospital Stay (Days) | | | | | | | | | | | | | | |
| 6 | Randomized trials | Serious^a^ | | Serious^b^ | Not serious | Serious^f^ | Serious^h^ | **◯◯◯**⨁ Very low | 235 | | 273 | **MD 0.03**  (-1.29 to 1.3) | 0.97 | 78 |
| ^a^ all were open label, and three trials lacked structured protocols for antiarrhythmic medications  ^b^ heterogeneity statistics is > 50% and not all confidence intervals overlap  ^f^ significant benefit or harm cannot be excluded  ^h^ not normally distributed but analyzed as such | | | | | | | | | | | | | | |
| Length of Intensive Care Unit Stay (Hours) | | | | | | | | | | | | | | |
| 3 | Randomized trials | Serious^i^ | | Not serious | Not serious | Serious^f^ | Serious^h^ | **◯◯◯**⨁ Very low | 143 | | 144 | **MD -2.07** (-16.19 to 12.05) | 0.77 | 47 |
| ^i^ all were open label, and two trials lacked structured protocols for antiarrhythmic medications  ^f^ significant benefit or harm cannot be excluded  ^h^ not normally distributed, but analyzed as such | | | | | | | | | | | | | | |
| Surgery Duration (Minutes) | | | | | | | | | | | | | | |
| 4 | Randomized trials | Serious^j^ | | Serious^e^ | Not serious | Serious^f^ | None | **◯◯◯**⨁ Very low | 183 | | 222 | **MD 16.06** (1.07 to 31.1) | 0.04 | 70 |
| ^j^ one trial excluded participants post-randomization  ^e^ heterogeneity statistics is > 50%  ^f^ significant benefit or harm cannot be excluded | | | | | | | | | | | | | | |
| Cross-Clamp Time (Minutes) | | | | | | | | | | | | | | |
| 4 | Randomized trials | Serious^j^ | | Serious^e^ | Not serious | Serious^f^ | None | **◯◯◯**⨁ Very low | 162 | | 164 | **MD 5.08**  (-2.44 to 12.6) | 0.19 | 76 |
| ^j^ one trial excluded participants post-randomization  ^e^ heterogeneity statistics is > 50% and not all confidence intervals overlap  ^f^ significant benefit or harm cannot be excluded | | | | | | | | | | | | | | |
| Cardiopulmonary Bypass Time (Minutes) | | | | | | | | | | | | | | |
| 5 | Randomized trials | Serious^k^ | | Not serious | Not serious | Not serious | None | **◯**⨁⨁⨁ Moderate | 212 | | 253 | **MD 12.89**  (7.37 to 18.41) | 0.00 | 43 |
| ^k^ two trial excluded participants post-randomization | | | | | | | | | | | | |  |  |
| Reoperations for Bleeding | | | | | | | | | | | | | | |
| 4 | Randomized trials | Serious^k^ | | Not serious | Not serious | Serious^f^ | None | **◯◯**⨁⨁ Low | 6/183  (3.3%) | | 4/219  (1.8%) | **RR 1.59** (0.48 to 5.28), -1.5% ARR | 0.45 | 0 |
| ^k^ two trials excluded participants post-randomization  ^f^ significant benefit or harm cannot be excluded | | | | | | | | | | | | | | |
| Stroke and/or Systemic Thromboembolism | | | | | | | | | | | | | | |
| 4 | Randomized trials | Serious^l^ | | Not serious | Not serious | Serious^f^ | None | **◯◯**⨁⨁ Low | 1/167  (0.6%) | | 2/165  (1.2%) | **RR 0.74** (0.14 to 3.87), 0.6% ARR | 0.72 | 0 |
| ^l^ all were open label, two trials lacked structured protocols for antiarrhythmic medications, and 3 lacked protocols for anticoagulant medications  ^f^ significant benefit or harm cannot be excluded | | | | | | | | | | | | | | |
| Mortality | | | | | | | | | | | | | | |
| 6 | Randomized trials | Serious^m^ | | Not serious | Serious^d^ | Serious^f^ | None | **◯◯◯**⨁ Very low | 2/298 (0.7%) | | 4/324  (1.2%) | **RR 0.76** (0.21 to 2.76), 0.5% ARR | 0.67 | 0 |
| ^m^ all were open label, two trials lacked structured protocols for antiarrhythmic medications  ^d^ trials had different definitions/different ascertainment techniques  ^f^ significant benefit or harm cannot be excluded | | | | | | | | | | | | | | |

**Appendix 10: Funnel Plots:**

**Early Post-operative AF:**

**
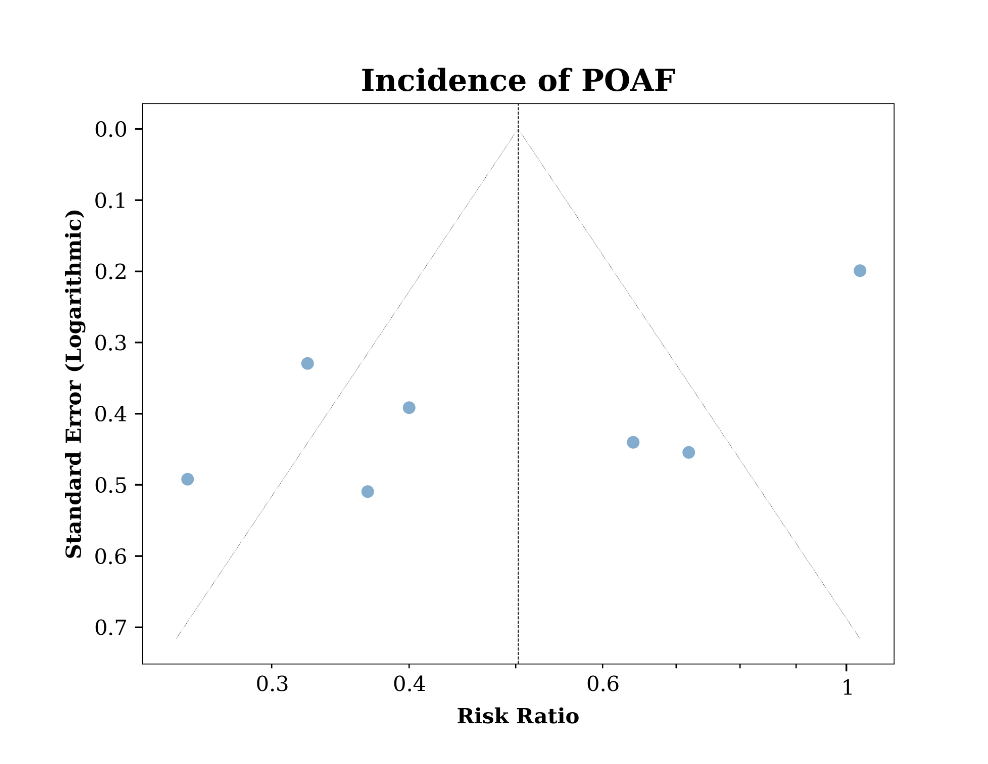
**

**Incident Clinical AF at Longest Follow-up:**

**
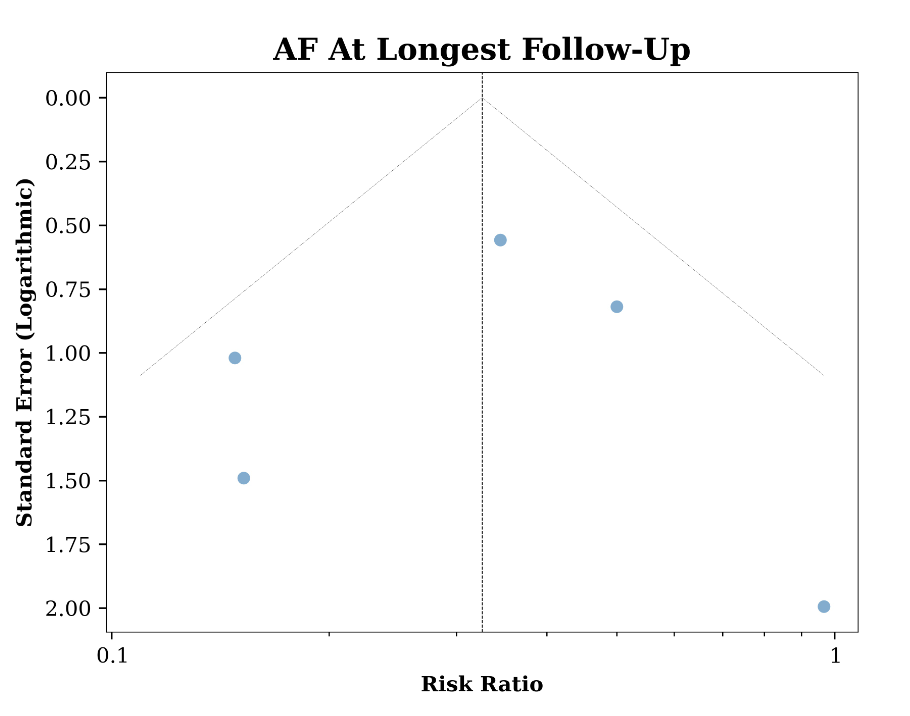
**

**Appendix 11: Description of Incident Clinical AF at Longest Follow-Up:**

| Trial: | Incident Clinical AF at Longest Follow-Up: |
| --- | --- |
| Al-Atassi 2014 | - Not reported |
| Kiaii 2015 | - AF assessed at 6 month follow-up - No patents experienced AF at long-term follow-up |
| Lednev 2017 | - Reported “1-year freedom from AF”   No information on whether patients experiencing AF at follow-up also experienced early post-operative AF |
| Revishvili 2020 | - Not reported |
| Revishvili 2023 | - Assessed “freedom from AF during 1-year follow-up” - No information on whether patients experiencing AF at follow-up also experienced early post-operative AF |
| Teijeira 2014 | - AF assessed at 24-month follow-up - All patients with AF at follow-up had AF in the post-operative period |
| Willekes 2023 | - Assessed AF during 1-year follow-up period - All patients with AF at follow-up had AF in the post-operative period |

**Appendix 12: Forest Plots for Other Outcomes:**

**Antiarrhythmic Use:
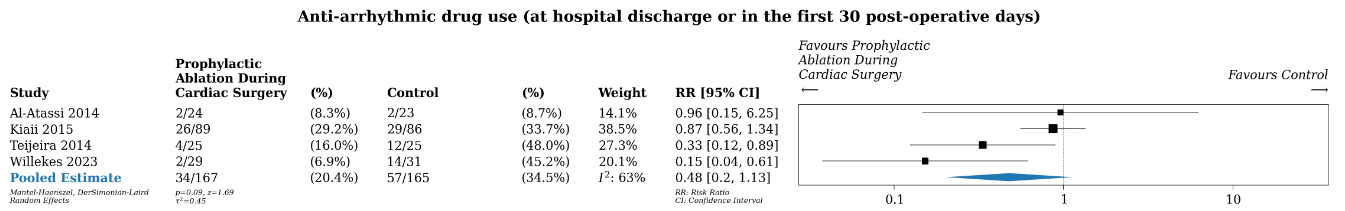
**

**Anticoagulant Use:**

**
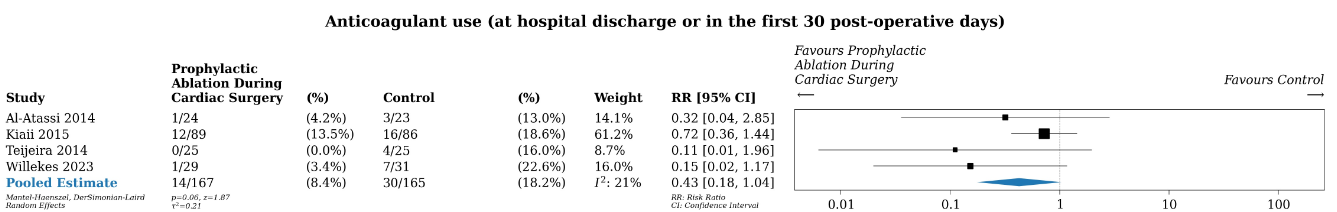
**

**Length of Hospital Stay (Days):**

**
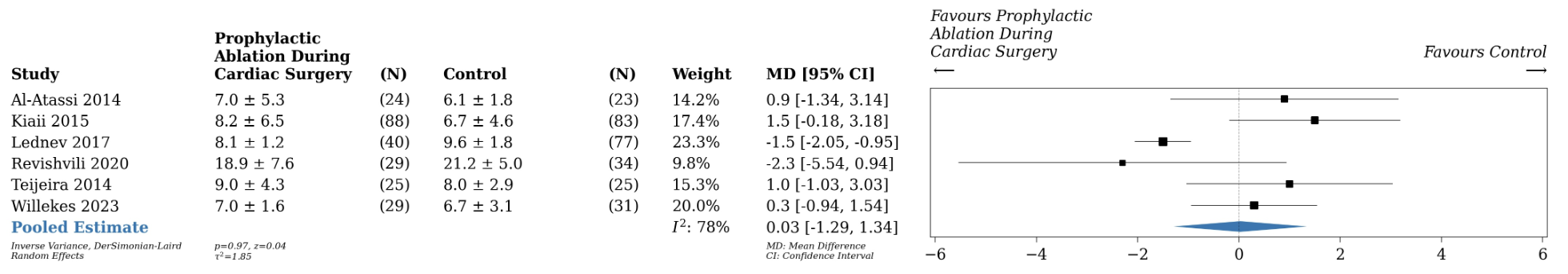
**

**Length of Intensive Care Unit Stay (Hours):**

**
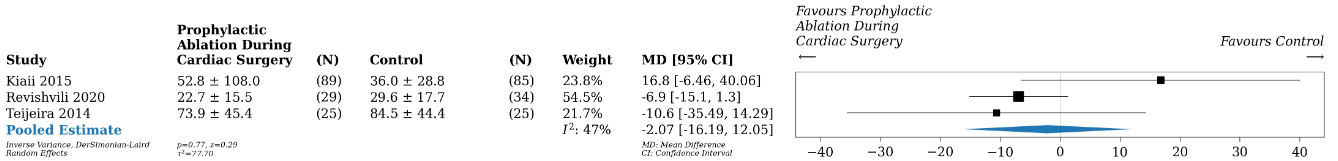
**

**Surgery duration (Minutes):**

**
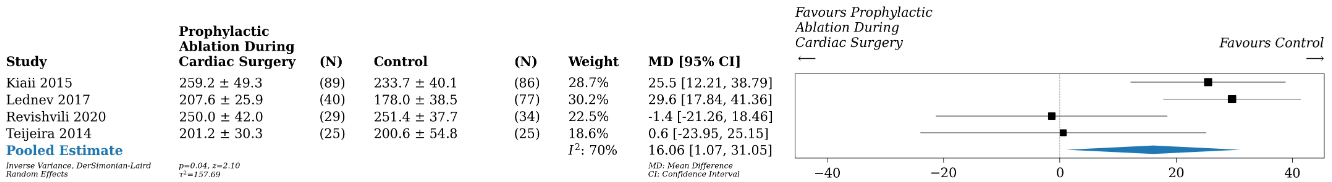
**

**Cross-clamp Time (Minutes):**

**
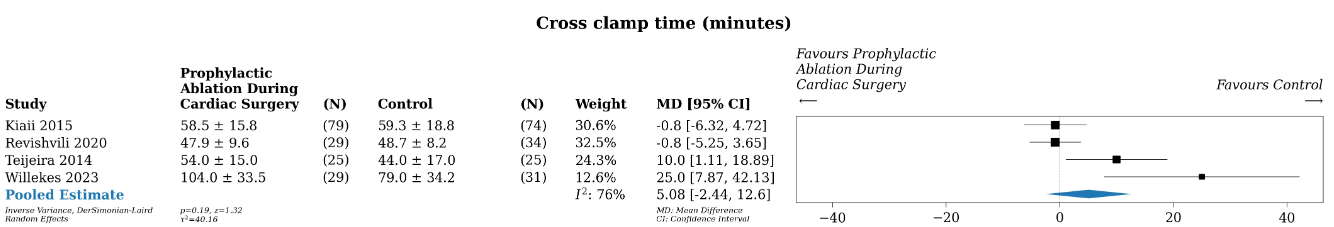
**

**Cardiopulmonary Bypass Time (Minutes):**

**
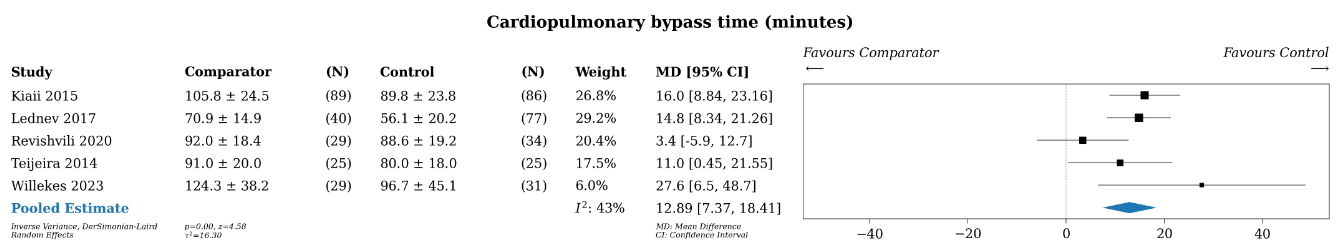
**

**Reoperations for Bleeding:**

**
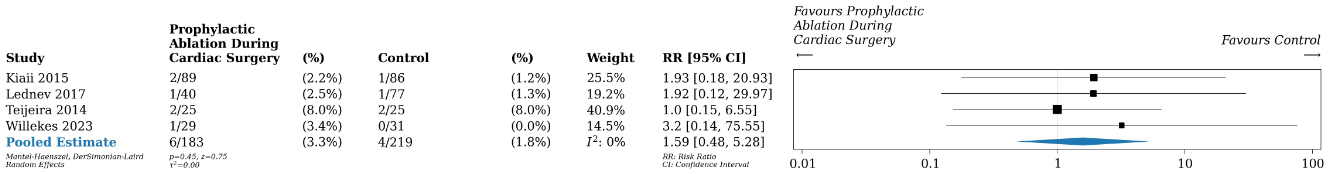
**

**Stroke and/or Systemic Thromboembolism:**


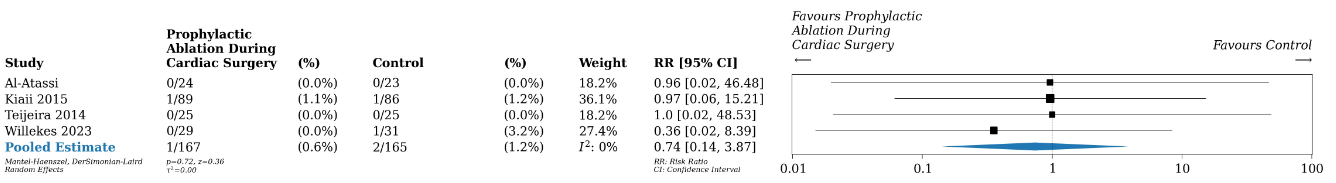


**Mortality:**

**
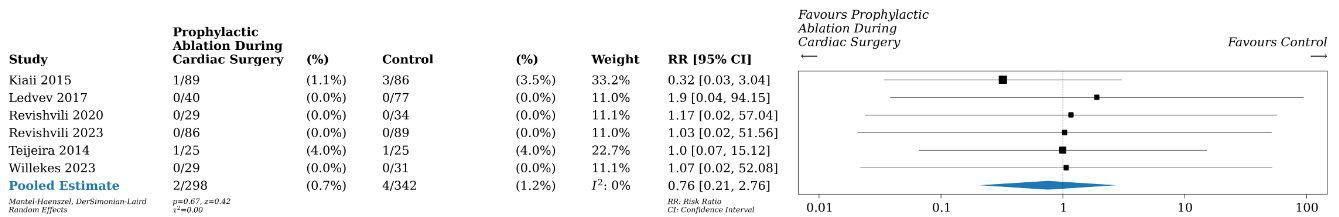
**

**Appendix 13: Description of Antiarrhythmic Use**

| Study: | Antiarrhythmic Use: |
| --- | --- |
| Al-Atassi 2014 | - Prescribed to patients post-operatively - Reported on as outcome |
| Kiaii 2015 | - Treatment for early post-operative AF - Reported on as an outcome |
| Lednev 2017 | - Patients in one arm of the trial received amiodarone prophylactically - Was not reported on as an outcome |
| Revishvili 2020 | - Treatment for early post-operative AF - Was not reported on as an outcome |
| Revishvili 2023 | - Patients in two arms of the trial received amiodarone prophylactically - Was not reported on as an outcome |
| Teijeira 2014 | - Prescribed to patients post-operatively - Reported on as outcome |
| Willekes 2023 | - Treatment for early post-operative AF - Reported on as an outcome |

1. 1 Absolute risk reduction [↑](#footnote-ref-1)
